# Long-term safety and dose escalation of intracerebroventricular CLN5 gene therapy in sheep supports clinical translation for CLN5 Batten disease

**DOI:** 10.3389/fgene.2023.1212228

**Published:** 2023-08-08

**Authors:** Nadia L. Mitchell, Samantha J. Murray, Martin P. Wellby, Graham K. Barrell, Katharina N. Russell, Ashley R. Deane, John R. Wynyard, Madeleine J. Palmer, Anila Pulickan, Phillipa M. Prendergast, Widler Casy, Steven J. Gray, David N. Palmer

**Affiliations:** ^1^ Faculty of Agriculture and Life Sciences, Lincoln University, Lincoln, New Zealand; ^2^ Department of Radiology, University of Otago, Christchurch, New Zealand; ^3^ Department of Pediatrics, University of Texas Southwestern Medical Center, Dallas, TX, United States

**Keywords:** neuronal ceroid lipofuscinosis, neurodegenerative disease, gene therapy, adeno-associated virus, intracerebroventricular, sheep

## Abstract

CLN5 neuronal ceroid lipofuscinosis (NCL, Batten disease) is a rare, inherited fatal neurodegenerative disorder caused by mutations in the *CLN5* gene. The disease is characterised by progressive neuronal loss, blindness, and premature death. There is no cure. This study evaluated the efficacy of intracerebroventricular (ICV) delivery of an adeno-associated viral vector encoding ovine *CLN5* (scAAV9/oCLN5) in a naturally occurring sheep model of CLN5 disease. CLN5 affected (CLN5^−/−^) sheep received low, moderate, or high doses of scAAV9/oCLN5 at three disease stages. The treatment delayed disease progression, extended survival and slowed stereotypical brain atrophy in most animals. Of note, one high dose treated animal only developed mild disease symptomology and survived to 60.1 months of age, triple the natural life expectancy of an untreated CLN5^−/−^ sheep. Eyesight was not preserved at any administration age or dosage. Histopathologic examination revealed that greater transduction efficiency was achieved through higher ICV doses, and this resulted in greater amelioration of disease pathology. Together with other pre-clinical data from CLN5^−/−^ sheep, the safety and efficacy data from these investigational new drug (IND)-enabling studies supported the initiation of the first in-human CLN5 gene therapy clinical study using the ICV delivery route for the treatment of CLN5 NCL.

**Clinical Trial Registration:** https://clinicaltrials.gov/, identifier NCT05228145

## 1 Introduction

The neuronal ceroid lipofuscinoses (NCLs, Batten disease) are a group of monogenic inherited lysosomal storage diseases characterized by progressive neurodegeneration, cortical atrophy, and blindness. Few effective treatments exist; the disease is presently fatal. Variant late-infantile NCL or CLN5 disease is caused by autosomal-recessive mutations in the *CLN5* gene. Whilst the function of this gene is unclear, it is known to encode a glycosylated, soluble non-enzymatic lysosomal protein (Isosomppi et al., 2002). Human CLN5 disease usually presents with motor dysfunction and impaired cognition between 1.5 and 8 years of age (Xin et al., 2010; Mole and Williams, 2013; Simonati et al., 2017), followed by progressive visual decline, seizures and dementia leading to premature death between the ages of 14–36 years (Nita et al., 2016; Simonati et al., 2017).

A naturally occurring sheep model of CLN5 NCL exists in New Zealand Borderdale sheep (Jolly et al., 2002; Frugier et al., 2008). Affected animals exhibit a human CLN5 disease-like phenotype including progressive brain atrophy and loss of vision from 11 to 12 months of age, and a shortened life span of <22 months (Jolly et al., 2002; Frugier et al., 2008). This sheep model has served as a powerful tool in the development and validation of a translational brain-directed CLN5 gene therapy product. For soluble lysosomal proteins, like CLN5, cross-correction of a small subset of neural cells is predicted to be sufficient to restore 5%–10% normal protein activity to facilitate normal cellular function (Sands and Davidson, 2006). Indeed, the single administration of brain-directed lentiviral or recombinant adeno-associated virus 9 (AAV9) vectors expressing ovine *CLN5* to CLN5 affected sheep resulted in long-term neurological disease attenuation (Mitchell et al., 2018). The intracerebroventricular (ICV) delivery route resulted in transduced corrected cells throughout the central nervous system, which were theoretically secreting CLN5 protein for uptake by neighbouring protein-deficient cells, thus providing surrounding cells and tissue with a continuous supply of soluble functional protein. This provided the first pressing case for translation of a CLN5 gene therapy product to human patients.

To further investigate the timing and optimal dose of an ICV treatment, cohorts of CLN5 affected sheep were treated with variable doses of recombinant self-complementary AAV9 vectors expressing codon-optimized ovine *CLN5* (scAAV9/oCLN5) at three different disease stages. Pre-symptomatic treatment was assessed in three cohorts of sheep at approximately 3 months of age at a low [LD; 8.0–8.2 × 10^10^ viral genomes (vg)] or moderate dose (MD, 2.4 × 10^11^ vg). Early symptomatic treatment was administered at 6–7 months of age with a similar MD (2.7 × 10^11^ vg) or high dose (HD; 2.8 × 10^12^ vg). Finally advanced symptomatic treatment was administered with a MD of 2.7 × 10^11^ vg of scAAV9/oCLN5 in 9-month-old CLN5 affected sheep. Sheep were regularly monitored until they were euthanized for humane reasons, at ages ranging from 18.5 to 60.1 months of age. The extensive in-life and neuropathological data from these cohorts provide further proof-of-concept for the gene therapy and delivery route and support its clinical translation. Importantly, this study was the first of two investigational new drug (IND)-enabling studies in sheep providing long-term and safety efficacy data on the CLN5 gene therapy product and useful translational data regarding the therapeutic window and effective ICV dosage.

## 2 Materials and methods

### 2.1 Animals

Supplementary Table S1 summarizes the study design. Clinically healthy CLN5 heterozygous (CLN5^+/−^) and CLN5 affected (CLN5^−/−^) Borderdale sheep were diagnosed at birth (Frugier et al., 2008) and maintained at Lincoln University under NIH guidelines for the Care and Use of Animals in Research and the NZ Animal Welfare Act (1999). The animal studies were reviewed and approved by the Lincoln University Animal Ethics and Institutional Biosafety Committees.

Sheep were randomized into treatment groups based on identification codes assigned to them at birth. Over a period of 3 years, nineteen CLN5^−/−^ ewes received bilateral intracerebroventricular (ICV) administration of a self-complementary adeno-associated virus serotype 9 vector expressing ovine *CLN5* (scAAV9/oCLN5) at various ages and dosage levels.

Treated sheep were euthanized by penetrating captive bolt to the cervical spine and immediate exsanguination at predetermined endpoints or when they significantly lost weight or condition, or their ovine Batten disease rating scale (oBDRS) scores approached 20. Concurrent age-matched CLN5^+/−^ and CLN5^−/−^ animals (*n =* 3–4 each) served as untreated healthy and affected controls respectively for each study. Data from these controls were pooled for the neurological examinations, whilst historic controls were used for the remainder of the in-life assessments and neuropathological analyses.

### 2.2 Vector and administration

Recombinant scAAV9, expressing codon-optimised ovine CLN5 (GenBank accession number NM_001082595; codon optimisation done by Atum bio, Menlo Park, CA, United States) under the control of the chicken beta actin (CBh) promoter (scAAV9/oCLN5), was produced by the University of North Carolina Gene Therapy Center Vector Core (NC, United States) by triple transfection of HEK293 cells, iodixanol gradient centrifugation and ion-exchange chromatography as described (Grieger et al., 2016). Vectors were formulated in 350 mM PBS containing 5% sorbitol, and titers determined by qualified droplet digital PCR.

Affected CLN5^−/−^ sheep received bilateral ICV injections using stereotactic surgical procedures as described previously (Linterman et al., 2011; Mitchell et al., 2018). Whilst doses differed, the vector was consistently delivered in a volume of 400 µL per hemisphere (total volume 800 µL), at a rate of 100 μL/min. Pre-symptomatic treatment was administered at 2.9–3.5 months of age at a low dose (LD; 8.0–8.2 × 10^10^ vg, *n =* 6) or moderate dose (MD; 2.4 × 10^11^ vg, *n =* 3). Early symptomatic treatment was administered at 5.9–7.5 months of age at a MD (2.67 × 10^11^ vg, *n =* 3) or high dose (HD; 2.8 × 10^12^, *n =* 4). Finally, advanced symptomatic treatment was administered at 8.7–9 months of age at a MD of 2.7 × 10^11^ (*n =* 3). The dosing regime allowed for direct comparison of a MD across the different timepoint interventions, whilst the LD treatment aimed to provide translational data on lowest effective clinical dose. Due to vector availability, the HD was only administered to early symptomatic sheep.

### 2.3 Neurological examination and electroretinography (ERG)

General physical health and neurological clinical assessments of the treated CLN5^−/−^ sheep and age-matched healthy control CLN5^+/−^ and untreated CLN5^−/−^ sheep were performed monthly by two independent blinded investigators. Scores for each of the ten parameters in the ovine Batten disease rating scale (oBDRS) ranged from 4 to 0 (normal to abnormal), giving a total score of 40 (Mitchell et al., 2018). Cranial nerve function, mentation, gait, head carriage and postural traits, manifest tremor or seizure onset were reported, as well as live weight data and body condition scores.

Mixed response ERG recordings were obtained from each eye of treated sheep as previously described (Murray et al., 2021). Dark adapted b-wave amplitudes were compared with historical data from healthy CLN5^+/−^ (*n =* 6) and untreated CLN5^−/−^ sheep (*n =* 6) (Russell et al., 2021).

### 2.4 Quantitative analysis of brain atrophy

Computed tomography (CT) scans were performed on treated sheep every 2 to 4 months (Russell et al., 2018). Helical slices were obtained at 1 mm intervals, 120 kV, 100 mA, 2 s rotation time on a GE HD750 CT scanner (GE Healthcare, Hyugo, Japan). Three-dimensional (3D) modelling and intracranial volumetrics were performed using the 3D slicer 4.3.1 freeware (http://www.slicer.org; RRID:SCR_005619) (Federov et al., 2012; Russell et al., 2018).

### 2.5 Immunohistochemistry

Sagittal sheep brain sections were stained for neuropathological markers and CLN5 expression (Oswald et al., 2005; Mitchell et al., 2018). In brief, 50 µm sections were blocked, 30 min with 1% H_2_O_2_ in PBS (pH 7.4) containing 0.3% Triton X-100 (PBST) and then 1 h in 15% normal goat serum (NGS) in PBST prior to overnight incubation in primary antibody diluted in 10% NGS in PBST at 4°C. The following primary antibodies were used: polyclonal glial fibrillary acidic protein (GFAP, 1:5,000; Agilent Cat# Z0334, RRID:AB_10013382) to detect activated astrocytes; a biotinylated form of the α-D-galactose specific isolectin I-B4 from *Griffonia simplicifolia* (GSB4, 1:500; Vector Laboratories Cat# B-1205, RRID:AB_2314661) for activated microglia and rabbit anti-sheep CLN5 (1:500; Viraquest Inc. Cat# R19122) for CLN5 protein expression. Immunoreactivity was detected using a biotinylated goat anti-rabbit IgG secondary antibody (1:1,000, Sigma-Aldrich Cat# B7389, RRID:AB_258613) for 4 h at room temperature, followed by ExtrAvidin peroxidase (1:1,000, Sigma-Aldrich Cat# E2886, RRID:AB_2620165) for 4 h. Staining was visualized by incubation in 0.5 mg/mL 3,3′-diaminobenzadine (DAB; Sigma-Aldrich Cat# D5637) and 0.01% H_2_O_2_ in PBS. Sections were mounted in a solution of 0.5% gelatine and 0.05% chromium potassium sulphate on glass slides, air-dried, dehydrated in 100% ethanol, cleared in xylene and coverslips mounted with DPX (BDH Chemicals, Poole, England).

A standard cresyl violet Nissl stain was used on adjacent sections to detect neuronal cytoarchitecture (Oswald et al., 2005). A parallel set of unstained sections was mounted, air-dried, and coverslipped with glycerol for fluorescent storage body analysis. Historical 50 µm brain sections from healthy control CLN5^+/−^ (*n =* 3) and untreated CLN5^−/−^ (*n* = 3) sheep were included in all staining runs as negative and positive controls.

### 2.6 Microscopy

Digital images of CLN5, GFAP, GSB4, Nissl-stained and unstained brain sections were obtained with a Nikon Digital Sight DSFi1 camera attached to a Nikon Eclipse 50i model microscope (Nikon Instruments Inc., Tokyo, Japan) utilizing NIS-Elements Software (Nikon Instruments; RRID:SCR_014329).

The number of CLN5-positive somata and fibres were semi-quantified (from zero to 100 CLN5-positive somata/0.57 mm^2^ field) in 20 fields for 11 distinct brain regions within each treated sheep hemisphere. At least 25 thickness measurements were taken through three different cortical regions and the cerebellum on Nissl-stained sections (Oswald et al., 2005). A set of 10 non-overlapping red-green-blue images were captured at each sampling site per hemisphere for each immunostain or with a 450–490 nm excitation/510 nm emission filter set for observation of fluorescent storage bodies.

Images were analyzed with ImageJ software (version 1.52p; National Institutes of Health (NIH), Bethesda, MD, United States; RRID:SCR_003070). Red bandwidth filters were applied, and a constant threshold used for each image set to select positively stained structures at low reactivity, but not background staining in regions of high reactivity. Results are presented as the percentage of immunoreactivity or fluorescence per sampled area.

### 2.7 *In vitro* analyses of CLN5 vector constructs

In preparation for clinical translation, a human version of the oCLN5 vector plasmid was generated by synthesizing a codon-optimized human CLN5 (*hCLN5*) transgene (Atum bio, Menlo Park, CA, United States) and replacing the *oCLN5* transgene with the *hCLN5* transgene. The plasmid was further modified to replace the ampicillin resistance gene on the bacterial backbone with the kanamycin resistance gene, to produce the final plasmid pSJGk-CBh-hCLN5opt-BGHpA. Transfection experiments were conducted to compare the expression of oCLN5 to hCLN5, with a CBh-EGFP (enhanced green fluorescent protein) plasmid used as a control. Transfections of either 1 µg or 2 µg of each plasmid were carried out using PEIpro (Polyplus, New York, NY, United States) in HEK293 cells (ATCC Cat# CRL-1573, RRID:CVCL_0045) in 6-well tissue culture plates. Cells were incubated for 48 h in 5% CO_2_ at 37°C, then cell lysates were subjected to Western blot analysis. The following antibody combinations were used: rabbit anti-human CLN5 primary antibody (Abcam Cat# ab170899) followed by goat anti-Rabbit IgG (H + L) DyLight 488 secondary antibody (Thermo Fisher Scientific Cat# A-11008, RRID:AB_143165), or mouse anti-GAPDH primary antibody (Abcam Cat# ab8245, RRID:AB_2107448) followed by IRDye 800CW goat anti-mouse IgG secondary antibody (LI-COR Biosciences Cat# 926-32210, RRID:AB_621842). Each experiment was performed 3 times and ImageJ software was used to quantify the bands. The intensity of the CLN5 band for each sample was normalized to the corresponding GAPDH band for further statistical analysis.

### 2.8 Statistical analysis

All statistical analysis was performed on GraphPad Prism software (v 9.0.0, GraphPad, La Jolla, CA, United States; RRID:SCR_002798). For the *in vitro* studies, one-way ANOVA was performed to assess the statistical significance levels amongst the 6 groups. For the neuropathological studies, means (% area stained, cortical thickness) and the corresponding standard errors of the mean (SEM) were computed for each brain region for each animal. These are presented in Supplementary Tables S2–S5. The means were used in a one-way ANOVA followed by multiple unpaired *t-*tests assuming unequal variances and using a false discovery approach with the two-stage linear step-up procedure of Benjamini, Krieger and Tekutieli with a Q = 0.05 to test each region separately for differences between control, treated and untreated CLN5^−/−^ cohorts.

## 3 Results

### 3.1 *In vivo* vector delivery

Bilateral ICV delivery was possible for all animals in the study, except an early symptomatic animal (1172–15) who received its total dose via sequential (unilateral) injections into the right lateral cerebral ventricle as it was not possible to locate the left ventricle. Data collected for more than 52 months post-ICV treatment show that the treatment was well tolerated, and no safety concerns were identified with any dose.

### 3.2 Survival

**FIGURE 1:**
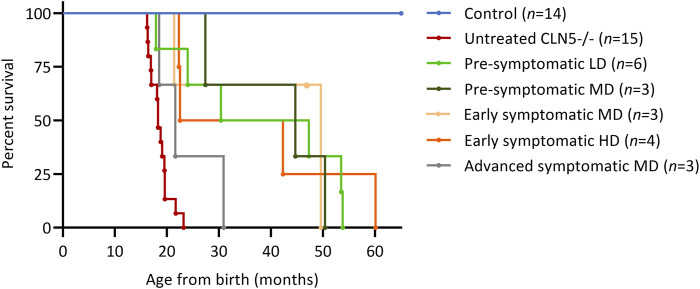
ICV scAAV9/oCLN5 greatly improves survival. Kaplan-Meier curves show survival was extended for ICV treated CLN5^−/−^ sheep when compared with healthy control CLN5^+/−^ (blue) and untreated CLN5^−/−^ (red) sheep. Treatment ages were pre-symptomatic (green), early symptomatic (orange) or advanced symptomatic (grey) whilst doses were low (LD), moderate (MD) or high dose (HD).

Several parameters were surveyed longitudinally in the nineteen treated sheep. These were survival, live weight, clinical score, visual function and intracranial volume. Fourteen of the sheep benefitted from the treatment, until they were euthanized at a pre-defined humane endpoint at 22.3–60.1 months of age. Survival was extended for at least twelve animals, and many doubled or tripled their humane life expectancy (Figure 1). The longest surviving sheep was in the early symptomatic HD cohort (1164–15), but this animal died unexpectedly at 60.1 months of age. A post-mortem examination revealed septicemia caused by gastrointestinal stasis. One animal in the second pre-symptomatic LD cohort (1115–18) was euthanized on welfare grounds at 17.9 months of age due to suspected acute polioencephalomalacia (PEM). This occurred 15 months after treatment and was not directly attributable to the gene therapy vector, surgical or administration procedure.

### 3.3 Live weight

Seven of the nine pre-symptomatic treated sheep gained weights equivalent to healthy control CLN5^+/−^ sheep, reaching adult weights of at least 70 kg and showing typical seasonal fluctuations due to fleece growth, before losing weight prior to euthanasia (Supplementary Figure S1). The early symptomatic treated sheep were generally lighter than their untreated CLN5^−/−^ cohorts at enrolment but continued to gain weight at 14 months of age, when the untreated CLN5^−/−^ weights began to plateau and the four treated animals still alive over 30 months reached similar adult weights to healthy control CLN5^+/−^ animals. Like the pre-symptomatic cohort, they too lost weight prior to euthanasia. The advanced symptomatic treated sheep that responded best to treatment (1163–16) ultimately reached a healthy adult weight of 77 kg which it maintained until its euthanasia at 30.9 months of age, however another animal in this treatment cohort (1143–16) failed to thrive.

### 3.4 Clinical signs and visual function

**FIGURE 2:**
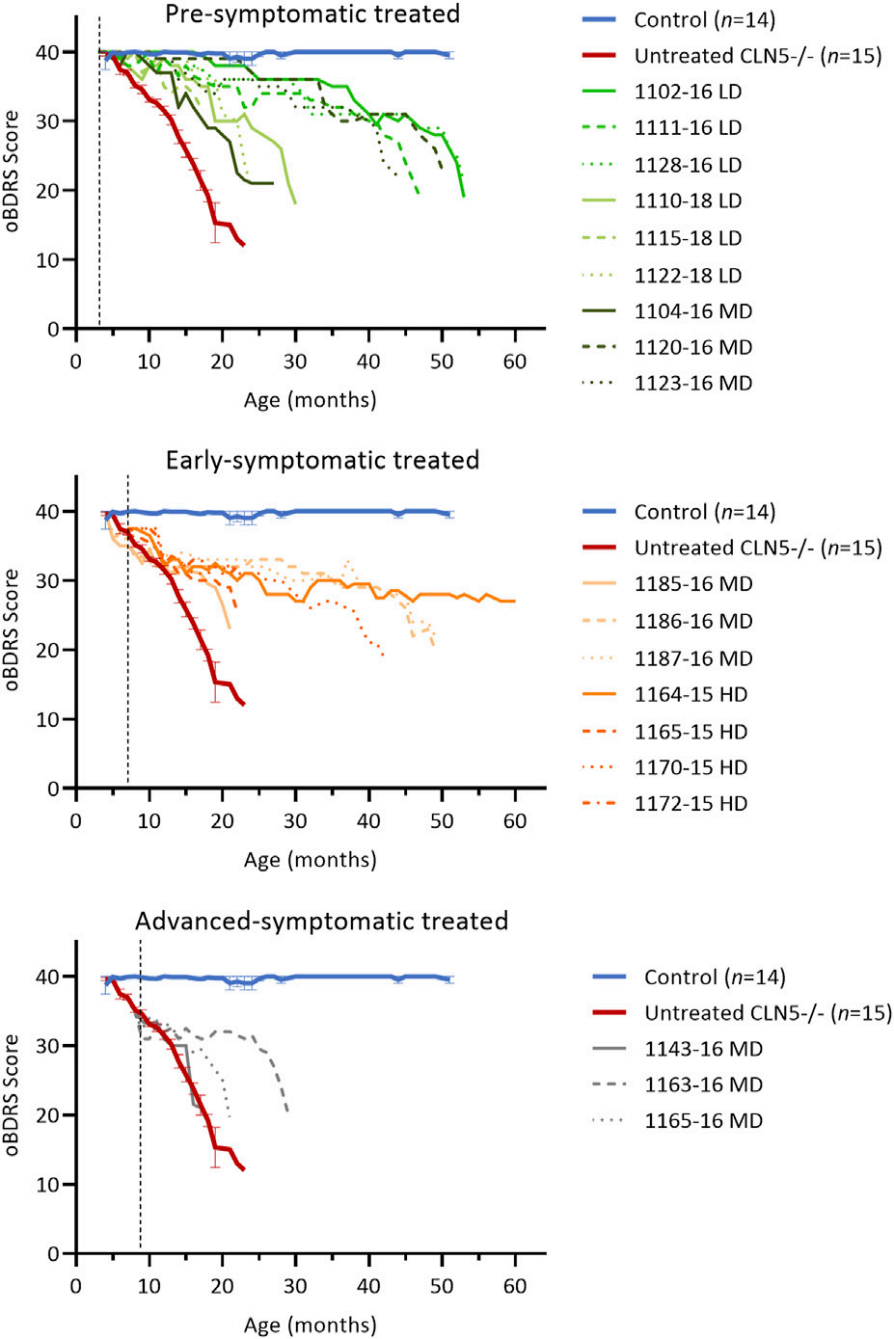
ICV scAAV9/oCLN5 delays the onset of clinical disease. Individual oBDRS changes in ICV treated CLN5^−/−^ sheep were compared with average data (±SEM) from healthy control CLN5^+/−^ (blue) and untreated CLN5^−/−^ (red) sheep. Disease progression was slowed for most treated sheep. Scores are displayed by treatment group (pre-symptomatic, green; early symptomatic, orange; advanced symptomatic, grey) at low (LD), moderate (MD) or high (HD) doses. Control data (*n =* 2–4) was collected concurrently with each study and pooled for presentation. Dashed lines indicate treatment age.

The ovine Batten disease rating scale (oBDRS) (Mitchell et al., 2018) was used to clinically assess treated sheep (Figure 2). Fifteen concurrent age-matched untreated CLN5^−/−^ sheep demonstrated the natural progressive disease course and were euthanized at 18.6 ± 0.5 months of age, with an average oBDRS score of 18.8 and rate of decline of 1.7 oBDRS units per month. Twelve age-matched healthy control CLN5^+/−^ sheep maintained oBDRS scores of 40 for the duration of the study.

Clinical disease was delayed for seven of the nine pre-symptomatic ICV treated sheep, although they all lost vision and ultimately succumbed to the disease (Figure 2; Supplementary Figure S2). The five animals who survived into their third or fourth years of life had rates of decline of 0.4–0.5 oBDRS units per month. For most of their lives, they only exhibited episodes of mild stereotypical behaviour, self-segregation, somnolence and reduced mentation (particularly MD animal 1123–16). It was not until the last 6 months of their lives that they demonstrated the wide stance, manifest ataxia and hind-limb paresis or localised seizure activity that is typically seen in the advanced stages of ovine CLN5 disease. Two further sheep (LD 1110–18 and MD 1104–16) were euthanised at 30.4 and 27.4 months of age with less delayed clinical disease, whilst a third sheep demonstrated increasing akinesia, inducible tremors, somnolence, and compromised sustainability in the field prompting humane euthanasia at 24 months of age (LD 1122–18). The other sheep in this cohort (LD 1115–18) only had mild clinical decline but was euthanized prematurely with suspected PEM.

Early symptomatic ICV treatment also significantly slowed further disease progression in five of the seven animals (Figure 2) but did not prevent vision loss (Supplementary Figure S2). From 1 year of age, there was no dramatic advance in motor, neurological or behavioural dysfunction in these animals and their oBDRS scores plateaued at approximately 30. During this time, these sheep were fully capable in the field, apart from occasional episodes of somnolence, reduced mentation, or mild inducible tics. Like the pre-symptomatic treated sheep, rates of decline ranged from 0.2 to 0.8 oBDRS units per month from treatment. Of note, HD-treated sheep 1170–15 was humanely euthanised with moderate disease symptoms at 42.3 months of age, double the maximal life expectancy of an untreated CLN5^−/−^ sheep, whilst HD-treated sheep 1164–15 survived even longer (60.1 months of age). The latter still had an oBDRS score of 27 at the time of her unexpected death and, whilst she exhibited mild episodes of stereotypical behaviour, ataxia and stress-inducible seizure activity, she maintained outdoor independence within the flock.

Advanced symptomatic sheep had an overt clinical phenotype prior to ICV treatment with scAAV9/oCLN5. They possessed a low head carriage, asymmetric reduction in menace (blink) response, depressed pupillary light reflex and reduced electroretinography response. Their herding instinct was diminished, and they tended to self-segregate, grazing alone in the field. The ICV treatment significantly slowed disease progression in one of the three treated animals (MD 1163–16) (Figure 2). After losing vision, the oBDRS score for this animal plateaued at 30 until 28 months of age (19 months post-treatment). Increasing episodes of inducible tremors, reduced mentation, stereotypical behaviour (circling in the field) and ultimately ataxia prompted humane euthanasia at 30.9 months of age, with an oBDRS score of 20. Clinical disease progression was only slightly delayed for a second animal in this cohort (MD 1165–16) whilst the third (MD 1143–16) was a non-responder.

### 3.5 Intracranial volume

**FIGURE 3:**
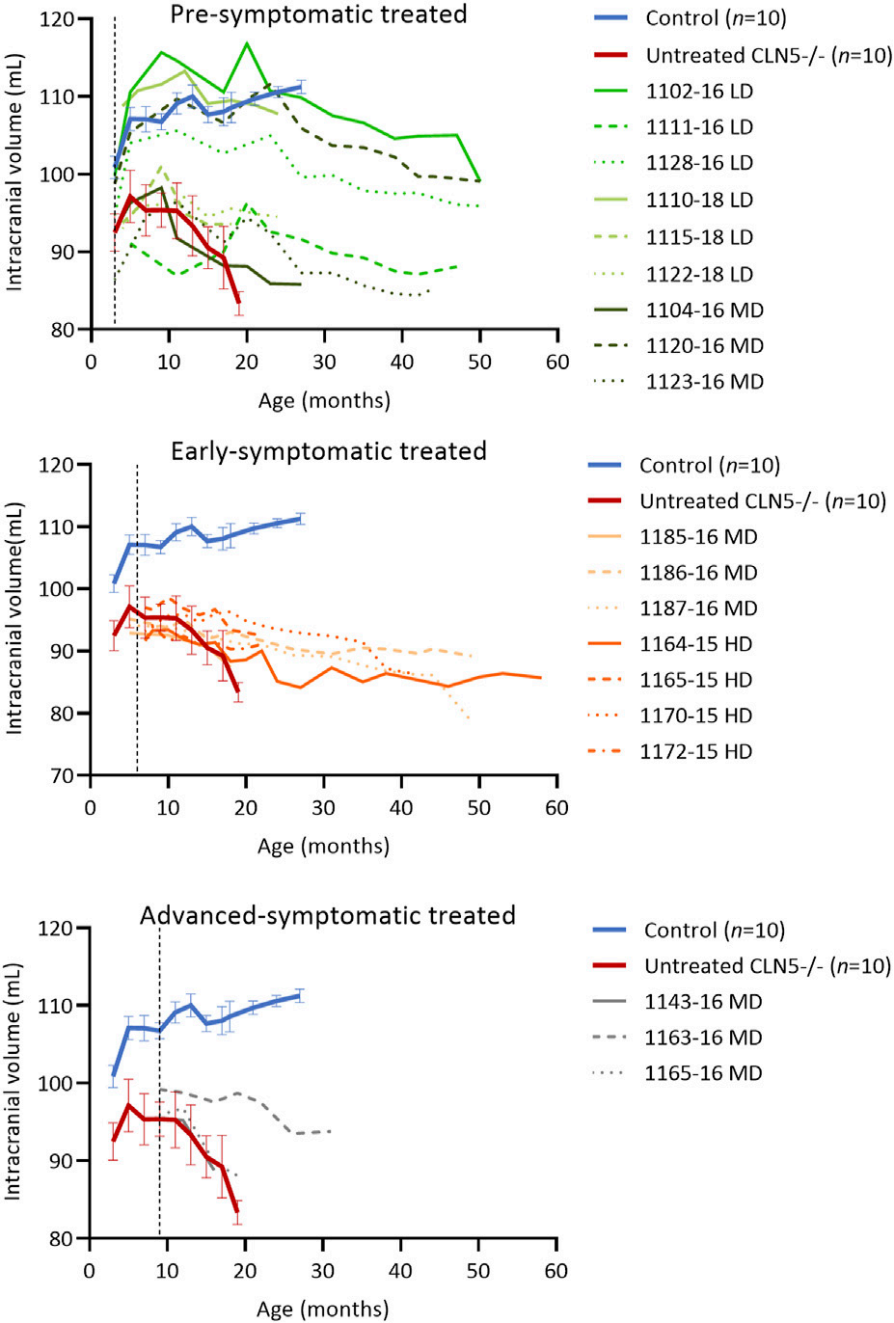
ICV scAAV9/oCLN5 delays intracranial volume loss. Individual intracranial volume changes in ICV treated CLN5^−/−^ sheep were compared with average data (±SEM) from healthy control CLN5^+/−^ (blue) and untreated CLN5^−/−^ (red) sheep. The ICV treatment slowed intracranial volume loss in most treated sheep, particularly when administered early in the disease course. Volumes are displayed by treatment group (pre-symptomatic, green; early symptomatic, orange; advanced symptomatic, grey) at low (LD), moderate (MD) or high (HD) doses. Dashed lines indicate treatment age.

Longitudinal computed tomography (CT) scanning (Russell et al., 2018) showed that the ICV treatment delayed intracranial volume loss, particularly when administered early in the disease course. Four of the pre-symptomatic treated sheep (LD 1102–16 and 1128–16, LD 1110–18, MD 1120–16) attained normal intracranial volumes of greater than 100 mL, similar to healthy control CLN5^+/−^ sheep, yet their general trend was a slow decline over time (Figure 3). The remaining pre-symptomatic treated sheep had intracranial volumes in the untreated CLN5^−/−^ range, although their rate of volume decline was slower, losing between 0.1 and 7.0 mL over their lifetime. For instance, MD-treated sheep 1104–16 had the greatest intracranial volume loss of 7.0 mL between ICV injection at 3 months, whilst untreated CLN5^−/−^ sheep lost on average 9.4 mL of intracranial volume between 3 and 19 months of age.

Early symptomatic treated sheep had intracranial volumes similar to, or less than, untreated CLN5^−/−^ sheep prior to ICV injection, yet post-injection brain atrophy was significantly slowed. Six of the seven treated sheep lost between 0.6 and 7.0 mL of intracranial volume, much less than untreated CLN5^−/−^ sheep lost over a shorter time. MD-treated sheep 1187–16 did have a larger loss of intracranial volume (15.4 mL) than untreated CLN5^−/−^ sheep, but again this was over a longer time (6.0–49.6 months of age).

Quantification of intracranial volume in advanced symptomatic sheep was consistent with clinical findings. Losses for two of the three sheep in this cohort were in line with untreated CLN5^−/−^ sheep, whilst the intracranial volume of the third sheep (MD 1163–16) declined only mildly to a terminal volume of 93.8 mL at 30.9 months of age. This was approximately 89% that of a 19-month-old healthy control CLN5^+/−^ volume.

### 3.6 CLN5 biodistribution

**FIGURE 4:**
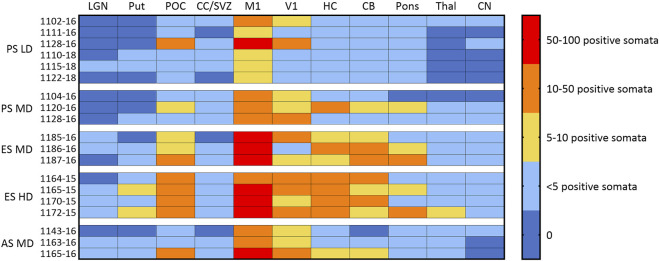
Ovine CLN5 protein is expressed in the brains of AAV9/oCLN5 treated sheep in a dose-dependent manner. The number of CLN5-positive somata and fibres were semi-quantified for 11 distinct brain regions in sheep treated intracerebroventricularly with scAAV9/oCLN5 at 3 (pre-symptomatic), 6 (early symptomatic), or 9 (advanced symptomatic) months of age. The ICV treatment predominantly targeted cortical structures and higher doses resulted in more transduced cells and greater CLN5 biodistribution. LGN lateral geniculate nucleus, Put putamen; POC parieto-occipital cortex; CC/SVZ corpus callosum/subventricular zone; M1 primary motor cortex; V1 primary visual cortex; HC hippocampus; CB cerebellum; Thal thalamus; CN caudate nucleus. Age of treatment: PS Pre-symptomatic; ES Early symptomatic; AS Advanced symptomatic. Dose: HD, high dose; MD moderate dose; LD low dose.

Endogenous CLN5 protein was widely expressed throughout the healthy control CLN5^+/−^ brain, particularly in the cortical neurons, pyramidal cells of the hippocampus and cerebellar Purkinje cells. No CLN5 protein expression was seen in the untreated CLN5^−/−^ sheep brain tissue (Supplementary Figure S3). Punctate vector-driven CLN5 expression was observed in the treated sheep brains in a largely dose-dependent manner (Figure 4; Supplementary Figure S3). LD treated sheep had mild to moderate CLN5 expression through the primary motor and visual cortices, whilst it was sparse elsewhere in the brain. The MD transduced more cells across the cortex, and the HD gave an even greater spread across the cortical mantle and into the subcortex that was detectable long-term, even at 60.1 months of age (over 4 years after treatment) in sheep 1164–15 (Figure 4). Transduced cells were primarily of neuronal morphology and included larger cortical neurons in the visual cortex, branching layer V pyramidal motor neurons, cells of the hippocampal hilus and CA1 region, as well as Purkinje cells and dendritic trees in the cerebellum (Supplementary Figure S3). However, most large subcortical structures, such as the thalamus, putamen and caudate nucleus, showed very low, or no, levels of detectable CLN5 expression even with the higher ICV doses.

### 3.7 Cortical thickness

**FIGURE 5:**
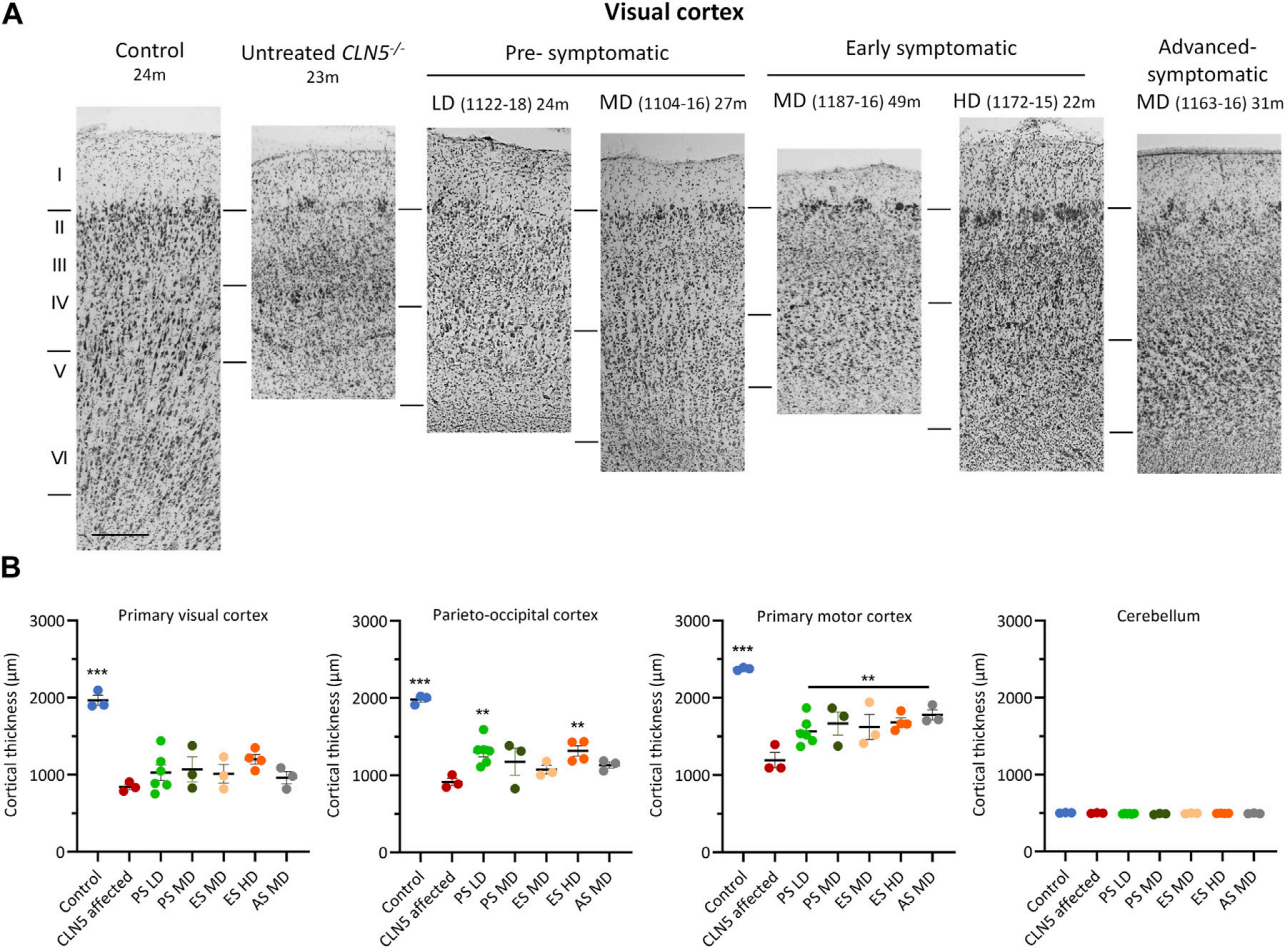
ICV scAAV9/oCLN5 attenuates cortical thinning. **(A)** Attenuation of cortical thinning can be seen in representative Nissl-stained images of the visual cortex of CLN5^−/−^ sheep treated intracerebroventricularly with scAAV9/oCLN5 at 3 (pre-symptomatic), 6 (early symptomatic), or 9 (advanced symptomatic) months of age when compared with healthy control CLN5^+/−^ and untreated CLN5^−/−^ sheep. The top line marks the layer I/II boundary, middle line indicates the layer IV/V boundary, and lower line denotes the layer VI/white matter boundary. Scale bar represents 200 µm. **(B)** Quantification of cortical or cerebellar thickness in four key regions which differentially undergo neurodegeneration in ovine CLN5 disease shows the treatment effect for individual animals in each group (PS pre-symptomatic, green; ES early symptomatic, orange; AS advanced symptomatic, grey). Significant differences to untreated CLN5^−/−^ are denoted by asterisks (***p < 0.001, **p < 0.01). Low dose (LD); moderate (MD); high (HD) dose.

A distinct laminar distribution of cells was observed across the cortical mantle of all ICV scAAV9/oCLN5-treated brains (Figure 5), in contrast to the marked atrophy and neuronal loss characteristic of the untreated CLN5^−/−^ cerebral cortex. However cortical thicknesses in the pre-symptomatic treated sheep were reduced compared with those of the healthy control CLN5^+/−^ brain, ranging from 61% to 79% of normal thickness in the treated motor cortex, and 38%–81% in the visual and parieto-occipital cortices albeit that most treated values were greater than those of much younger 24-month-old untreated CLN5^−/−^ animals (Supplementary Table S2).

The early symptomatic treatment also attenuated cortical thinning, with most treated thicknesses greater than untreated CLN5^−/−^ brains in all regions examined. This did not always reach statistical significance (Figure 5), but it should be noted that treated sheep brains were considerably older at euthanasia. Whilst a corrective effect on clinical status was not observed for all animals in the advanced symptomatic cohort, the treatment ameliorated their cortical thinning. The greatest treatment effect was observed in animal 1163–16, whose cortical thicknesses at 30.9 months of age were 1.3–1.6 times that of much younger untreated CLN5^−/−^ sheep (Figure 5; Supplementary Table S2).

Natural history studies have shown that the cerebellum does not significantly degenerate in CLN5^−/−^ sheep (Mitchell et al., 2023) and there was also no apparent cerebellar thinning in any treated sheep over the study (Figure 5; Supplementary Table S2).

### 3.8 Neuroinflammation

**FIGURE 6:**
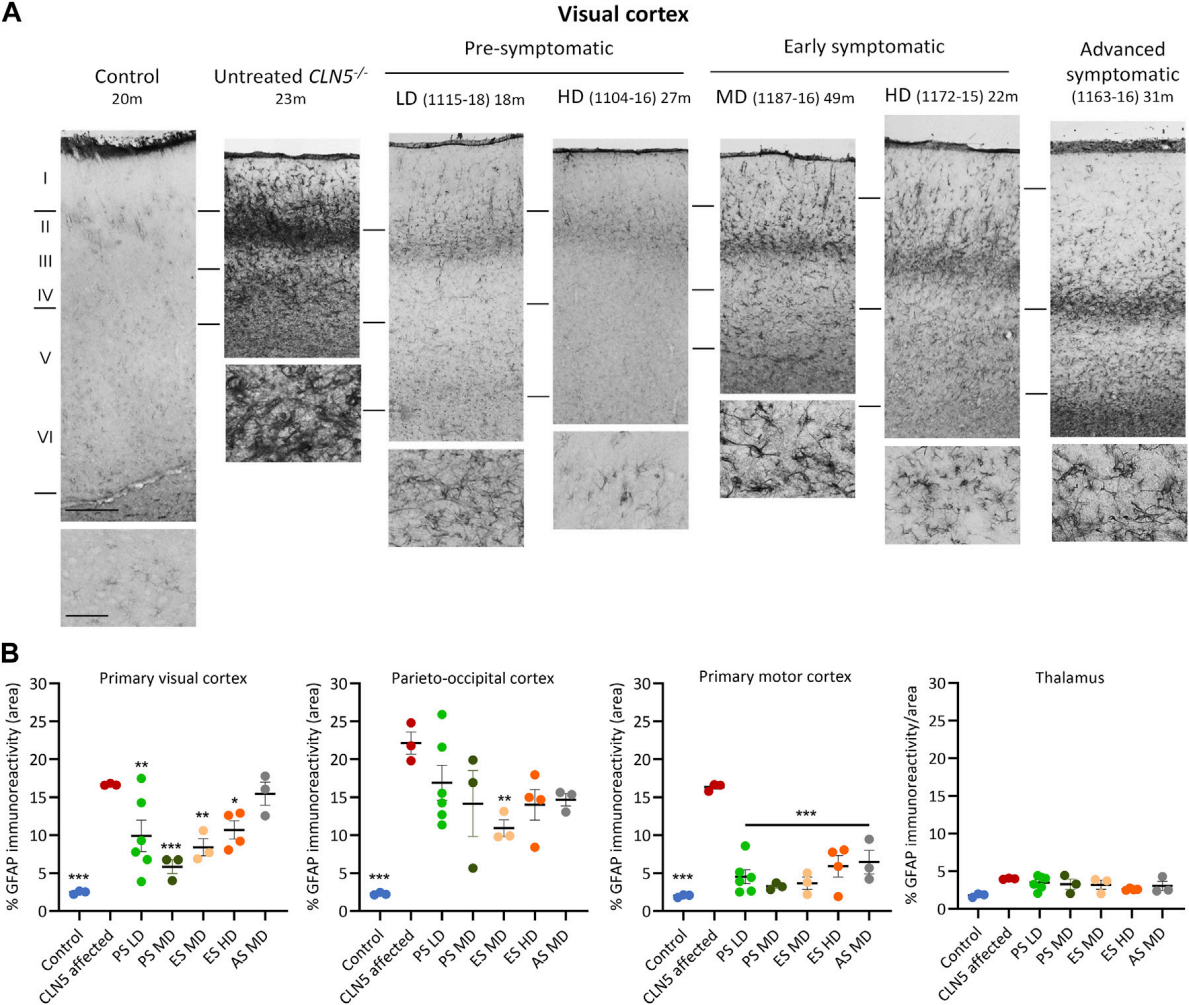
ICV scAAV9/oCLN5 attenuates astroglial activation. **(A)** The positive treatment effect on astrogliosis can be seen in representative GFAP-immunostained images of the visual cortex of CLN5^−/−^ sheep treated intracerebroventricularly with scAAV9/oCLN5 at 3 (pre-symptomatic), 6 (early symptomatic), or 9 (advanced symptomatic) months of age when compared with healthy control CLN5^+/−^ and untreated CLN5^−/−^ sheep. The top line marks the layer I/II boundary, middle line indicates the layer IV/V boundary, and lower line denotes the layer VI/white matter boundary. Scale bar represents 200 µm. **(B)** Quantification of astrocytosis in four key regions which differentially undergo neurodegeneration in ovine CLN5 disease shows the treatment effect for individual animals in each group (PS pre-symptomatic, green; ES early symptomatic, orange; AS advanced symptomatic, grey). Vertical bars indicate +SEM. Significant differences to untreated CLN5^−/−^ are denoted by asterisks (***p < 0.001, **p < 0.01, *p < 0.05). Low dose (LD); moderate (MD); high (HD) dose.

The neuroinflammatory response was assessed using an astrocytic marker (GFAP) and a microglial marker (GSB4). The dense glial meshwork of hypertrophic activated astrocytes across the upper cortical layers of the untreated CLN5^−/−^ brain was not present in most regions of the pre-symptomatic ICV treated brains, regardless of scAAV9/oCLN5 dose (Figure 6). Typically, astroglial morphologies in the younger treated brains resembled those seen in the healthy control CLN5^+/−^ brain, being fibrous in the white matter and highly branched and protoplasmic with numerous processes in the cortical grey matter (Figure 6A) but they had become activated in the symptomatic treated brains. A similar trend was observed with the GSB4 microglial marker (Supplementary Figure S4). Whilst there were clusters of GSB4-positive activated microglia and amoeboid brain macrophages in the untreated CLN5^−/−^ brain, there were fewer activated microglia in the cortical parenchyma of most of the pre-symptomatic treated sheep although this did not reach statistical significance.

By 6 months of age, neuroinflammation in the CLN5^−/−^ sheep brain is pronounced and spread across the cortical mantle, although the primary visual and parieto-occipital cortices remain the most affected regions (Mitchell et al., 2023). Both GFAP and GSB4 staining of early symptomatic treated brains indicated a halt or slowing of further astrocytosis, brain macrophage formation and recruitment. Although astrocytes were hypertrophied in the treated brains, the strong meshwork of activated astrocytes was not observed and total reactivity of both neuroinflammatory markers was often significantly less than that in untreated CLN5^−/−^ brains. In some instances, GFAP immunoreactivity in the treated motor cortex approached healthy control CLN5^+/−^ levels.

Gliosis would have been pronounced in the three advanced symptomatic treated animals’ brains at their time of ICV delivery (9 months). Treatment had no substantive effect in two animals of the cohort, but amelioration in disease-associated neuroinflammation was observed in the brain of animal 1163–16 (Supplementary Tables S3, S4). Glial activation within the brain of this animal was to a much lesser extent than without treatment.

To verify if reduced CLN5 transduction in the subcortex resulted in heightened neuroinflammation, GFAP and GSB4 reactivities were also assessed in the thalamus and cerebellum of ICV treated sheep. Although expression of both neuroinflammatory markers was elevated in the thalamus (Figure 6; Supplementary Figure S4), it did not significantly exceed that of much younger untreated CLN5^−/−^ sheep and was noticeably less than that observed in the cortical regions. There was no evidence of astrocytic or microglial activation in either the untreated CLN5^−/−^ or ICV treated cerebella (Supplementary Figure S5).

### 3.9 Lysosomal storage

**FIGURE 7:**
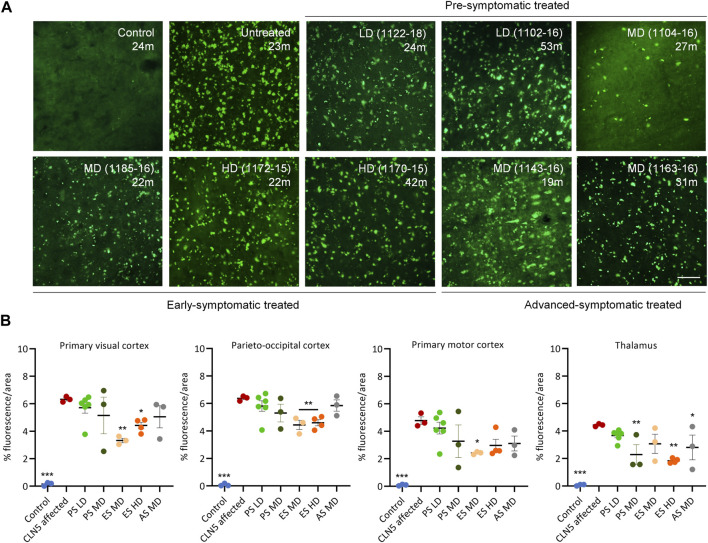
ICV scAAV9/oCLN5 slows lysosomal storage accumulation. **(A)** Lysosomal storage burden was reduced in most CLN5^−/−^ sheep treated intracerebroventricularly with scAAV9/oCLN5 at 3 (pre-symptomatic), 6 (early symptomatic), or 9 (advanced symptomatic) months of age when compared with healthy control CLN5^+/−^ and untreated CLN5^−/−^ sheep. Representative fluorescent images of storage material in the visual cortex show this effect. Scale bar represents 50 µm. **(B)** Quantification of the mean percentage area of fluorescence in four key regions which differentially undergo neurodegeneration in ovine CLN5 disease shows the treatment effect for individual animals in each group (PS pre-symptomatic, green; ES early symptomatic, orange; AS advanced symptomatic, grey). Vertical bars indicate +SEM. Significant differences to untreated CLN5^−/−^ are denoted by asterisks (***p < 0.001, **p > 0.01, *p < 0.05). Low dose (LD); moderate (MD); high (HD) dose.

Punctate globular storage bodies were densely packed into the cortical neurons and cerebellar Purkinje cells of untreated CLN5^−/−^ sheep brains (Figure 7 **;** Supplementary Figure S6). There was less storage in one pre-symptomatic MD treated sheep (1104–16), but the LD or MD was insufficient to attenuate accumulation in the remaining animals.

Quantitative threshold analysis showed there was significantly less fluorescent material in the primary visual and parieto-occipital cortex of the early symptomatic treated brains compared with much younger CLN5^−/−^ sheep (Figure 7). Of note, HD-treated sheep 1164–15 and 1170–15 who were euthanized at 60.1 and 42.3 months of age respectively, had less than or similar storage levels to untreated CLN5^−/−^ animals nearly 2 years younger (Supplementary Table S5).

Storage body accumulation in the advanced symptomatic treated sheep paralleled neuroinflammation. Storage burden was significantly reduced in sheep 1163–16 compared with untreated CLN5^−/−^ sheep, particularly in the visual and motor cortices (Figure 7; Supplementary Table S5).

Although fewer transduced cells were detected in the subcortex of ICV treated sheep than cortical structures, this did not have a greater effect on subcortical lysosomal accumulation. Storage burden was attenuated in the thalamus and cerebellum for the majority of ICV treated sheep, and significantly reduced in the thalamus of MD treated sheep and the cerebellum and thalamus of HD treated sheep when compared with untreated CLN5^−/−^ sheep (Figure 7; Supplementary Figure S6).

### 3.10 *In vitro* vector analyses

Finally, in addition to an ovine CLN5 (*oCLN5*) construct generated for the *in vivo* studies in sheep, a parallel human CLN5 (*hCLN5*) construct was made for possible downstream human use. Both plasmid constructs were identical between the inverted terminal repeats (ITRs), except for the species specificity of the *CLN5* transgene. Both human and ovine genes underwent independent codon-optimizations. To confirm expression of the constructs and compare their relative strength of expression, both plasmids were transfected into HEK293 cells and protein levels assessed by Western blotting. While minimal endogenous CLN5 protein could be detected by Western blot under the experimental conditions, expression was readily apparent in a dose-responsive manner 48 h following transfection of either the hCLN5 or oCLN5 plasmid (Supplementary Table S7). When 2 ug of plasmid was transfected, the hCLN5 construct showed a 1.26-fold higher expression compared to oCLN5 (*p* = 0.002), whereas when 1 ug was transfected the hCLN5 showed 2.82-fold higher expression that was not statistically significant (*p* = 0.225).

## 4 Discussion

These studies provide pre-clinical proof-of-concept data which supported the successful United States Food and Drug Administration clearance of the CLN5 gene therapy product as an investigational new drug for the treatment of human CLN5 Batten disease. New Zealand Borderdale sheep with naturally occurring CLN5 NCL were used as they represent an ideal model in which to develop and test potential gene therapies for this largely pediatric condition. Nineteen CLN5^−/−^ sheep received ICV scAAV9/oCLN5 therapy at three different ages representing different stages of disease development and over a 35-fold range of doses. A summary of the results is presented in Table 1.

**TABLE 1:** Summary of in-life efficacy and neuropathological terminal endpoints following ICV scAAV9/oCLN5.

**Study**	**Treatment**	**ICV dose (vg)^a^**	**Sheep**	**Tx Age (m)**	**End point (m)**	**Clinical Description**	**Rate of Decline** ^b^	**Loss of vision:**	**Intracranial volume change (mL)** ^c^	**Terminal brain weight (g)**	**Cortical thickness^d^**	**Neuroinflammation**	**Lysosomal storage**	**CLN5 expression**
Pre-symptomatic	ICV scAAV9/oCLN5	LD/1 8.0x10^10^	1102–16	3.2	53.8	Delayed clinical disease	-0.4	Yes	-0.7	65.7	45-67%	High	Extensive	Mild to moderate in cortex, sparse remaining CNS
			1111–16	3.2	47.3	Delayed clinical disease	-0.5	Yes	-3.0	66.0	38-58%	High	Extensive	Moderate in M1, sparse remaining CNS
			1128–16	2.9	53.5	Delayed clinical disease	-0.4	Yes	1.2	73.5	44-67%	Moderate	Extensive	Moderate to extensive across cortex
		LD/2 8.2x10^10^	1110–18	3.5	30.4	Delayed clinical disease	-0.9	Yes	-1.0	72.4	59-70%	High	Extensive	Sparse in cortex
			1115–18	3.5	17.9	Euthanised with PEM	-0.7	Yes	-0.1	85.0	73-81%	Moderate	Extensive	Mild in M1, sparse remaining CNS
			1122–18	3.0	24.0	Moderate clinical disease	-1.1	Yes	1.5	74.8	53-67%	Moderate	Reduction	
		MD 2.4x10^11^	1104–16	3.2	27.4	Delayed clinical disease	-0.8	Yes	-7.0	65.4	70-79%	Low	Reduction	Mild across cortex
			1120–16	3.2	50.4	Delayed clinical disease	-0.4	Yes	0.3	82.4	51-74%	Moderate	Extensive	Moderate across cortex
			1123–16	2.9	44.7	Delayed clinical disease	-0.4	Yes	-1.2	59.0	42-58%	Moderate	Extensive	Moderate in M1 and V1
Early symptomatic	ICV scAAV9/oCLN5	MD 2.7x10^11^	1185–16	6.0	21.4	Moderate clinical disease	-0.8	Yes	-5.5	73.5	59-82%	Low	Reduction	Mild to extensive across cortex and cerebellum, very little in subcortex
			1186–16	6.0	49.6	Delayed clinical disease	-0.4	Yes	-6.0	74.0	50-64%	Low	Reduction	
			1187–16	6.0	49.6	Delayed clinical disease	-0.3	Yes	-15.4	75.1	42-60%	Moderate	Reduction	
		HD 2.8x10^12^	1164–15	7.5	60.1	Mild decline	-0.2	Yes	-6.0	78.3	60-67%	Moderate	Slight reduction	Mild to moderate across cortex and subcortex
			1165–15	7.5	22.5	Mild clinical disease	-0.7	Yes	-4.5	76.8	62-72%	Moderate	Reduction	Mild to extensive across cortex and cerebellum, very little in subcortex
			1170–15	7.3	42.3	Delayed clinical disease	-0.5	Yes	-7.0	73.9	54-77%	Low	Reduction	
			1172–15	7.3	22.3	Mild decline	-0.3	Yes	-0.6	73.5	73-77%	Moderate	Reduction	
Advanced symptomatic	ICV scAAV9/oCLN5	MD 2.7x10^11^	1143–16	9.0	18.5	Stereotypical clinical disease	-1.6	Yes	-6.7	67.9	50-72%	High	Extensive	Mild across cortex
			1163–16	8.8	30.9	Delayed clinical disease	-0.6	Yes	-5.4	71.8	56-80%	Low	Reduction	Mild to extensive across cortex and cerebellum, very little in subcortex
			1165–16	8.8	21.6	Moderate clinical disease	-1.1	Yes	-7.8	69.8	42-72%	High	Extensive	Mild across cortex
Control CLN5^+/-^	Nil		*n* = 12	N/A	>24	Normal	0.0	No	3.3	106.1 ± 1.5	100%	None	No present	Endogenous throughout CNS
Untreated CLN5^-/-^	Nil		*n* = 15	N/A	18.6	Stereotypical clinical disease	-1.7	Yes	-9.4	62.0 ± 1.0	37-42%	Extensive	Extensive	Absent

Abbreviations: CNS, central nervous system; HD, high dose; ICV, intracerebroventricular; m, months; M1 primary motor cortex; MD, moderate dose; N/A not applicable; oBDRS, ovine Batten disease rating scale; Tx Treatment.

^a^ Two low dose trials have been performed. The first was designated LD/1 and the second LD/2.

^b^ Rate of decline in oBDRS, units per month. For treated sheep this is from baseline (treatment age) to end point; for controls this is from 3 months to end point.

^c^ Total intracranial volume change from baseline (treatment age) to end point for treated sheep, or from 3 to 19 months of age for controls (Russell et al., 2018).

^d^ % cortical thickness of 24-month-old healthy control CLN5^+/−^

Theoretically the earlier gene therapy can be administered to patients with neuropathic lysosomal storage diseases, the more the clinical benefit. Three months of age is a pre-symptomatic disease stage in CLN5 affected sheep, modelling patients being symptom-free, with maximum functional neuronal cells present, and before cumulative pathogenic processes, including neuroinflammation and lysosomal storage body accumulation, become manifest. Given that a CLN5 diagnosis in humans can take years, the equivalent pre-symptomatic population in humans is normally restricted to younger siblings or close relatives of already diagnosed symptomatic CLN5 patients.

Nine *CLN5* ^*−/−*^ sheep have received pre-symptomatic ICV scAAV9/oCLN5 therapy at 3 months of age at two doses (8.0–8.2 × 10^10^ to 2.4 × 10^11^ vg). The treatment provided long-term therapeutic benefit. Clinical disease was considerably delayed and five of the treated animals survived to more than double the natural life expectancy of untreated CLN5^−/−^ sheep. Longitudinal neuroimaging indicated a halt or slowing in the stereotypical brain atrophy of ovine CLN5 disease and four of these sheep maintained intracranial volumes in the range of healthy CLN5^+/−^ controls before declining slowly over time. One pre-symptomatic treated sheep (1104–16) did not respond as positively to the moderate dose (2.4 × 10^11^ vg) ICV treatment and was euthanized at 27.4 months of age with developing clinical disease. Despite its 7.0 mL loss in intracranial volume, this was at a slower rate than untreated CLN5^−/−^ sheep and post-mortem analyses revealed a significant reduction in disease-associated neuropathology. The low level of CLN5 transduction efficiency in the brain of this animal is likely causative of the reduced therapeutic benefit compared with her similarly treated cohorts.

One of the pre-symptomatically treated sheep (LD 1115–18) was euthanized on welfare grounds at 17.9 months of age due to suspected acute polioencephalomalacia (PEM). This neurological disease, also known as cerebrocortical necrosis, is seen worldwide in ruminants and presents sub-acutely with similar clinical signs to ovine Batten disease hence diagnosis was not detected. PEM is primarily associated with thiamine or dietary deficiencies (Lévy, 2020) but no cause was identified in this case and no other animals in the flock developed the condition. The brain of sheep LD 1115–18 lacked the definitive post-mortem tests for PEM, faint yellow discoloration and/or fluorescence of the grey matter under ultraviolet light, so diagnosis could not be confirmed. However, it occurred 15 months after treatment, thus there was no evidence to attribute this condition to the gene therapy vector, anaesthesia, surgical or administration procedure.

These results in pre-symptomatic sheep correlate well with previous CLN5 gene therapy studies in the Borderdale sheep model. Both single-stranded AAV9 and lentiviral vectors expressing ovine *CLN5* have been shown to normalize intracranial volume and prevent disease onset and progression when delivered to CLN5^−/−^ sheep at 3 months of age (Mitchell et al., 2018). This study showed that the current central nervous system (CNS) viral vector of choice, self-complementary AAV9, can also be added to the suite of therapeutic vector platforms for CLN5 disease.

Most human cases of Batten disease only become diagnosed following the development of overt disease, represented by the six-month-old early symptomatic CLN5^−/−^ cohort. At this age, affected sheep demonstrate mild movement dysfunction and early visual loss, and cortical atrophy and neurodegeneration is obvious. Seven CLN5^−/−^ sheep received moderate (2.7 × 10^11^ vg) or high dose (2.8 × 10^12^ vg) ICV scAAV9/oCLN5 therapy between six and 7 months of age. The treatment slowed disease progression in most treated animals. Live weight largely normalized, quality of life was improved, and lifespan extended to more than double to triple the natural expectancy of CLN5^−/−^ sheep. Three animals survived to be over 42 months of age, and a fourth (1164–15) until over 60 months when it died unexpectedly of other causes. Aside from loss of vision, these sheep showed only mild phenotypic changes long after ICV injection. In particular, sheep 1164–15 had only mild episodes of stereotypical behavior and ataxia at her untimely death and did not show manifest ataxia, localized seizure activity or require extensive nursing. Intracranial volumes at injection were equivalent to those of untreated CLN5^−/−^ sheep, and losses observed over the duration of the study occurred much more slowly than they do in untreated CLN5^−/−^ sheep.

Amelioration of all disease-related neuropathological markers indicated long-term treatment efficacy, albeit not complete. The treated sheep with the least therapeutic efficacy (MD 1185–6) was the animal with the lowest number of transduced cells, whilst there were many CLN5-positive cells in the motor, visual and parieto-occipital cortices, hippocampus and cerebellum of the HD early symptomatic treated sheep who survived to 60.1 months of age, indicating some linkage between transduction efficiency and therapeutic efficacy.

Most human CLN5 cases are of a variant late infantile onset form, presenting between 1.5 and 8 years of age (Xin et al., 2010; Mole and Williams, 2013; Simonati et al., 2017), then progressing to severe impairment in the learning/cognition, motor, language, vision, and seizures domains and premature death. The advanced symptomatic nine-month-old CLN5^−/−^ cohort was studied to determine the effectiveness of a treatment at this stage. At this age, untreated CLN5^−/−^ sheep demonstrate clear phenotypic deficits, particularly related to visual loss, and the affected brain has reduced to approximately 85% the weight of a healthy sheep brain (Mitchell et al., 2023). Neuronal loss is advanced, particularly from the occipital cortex where cortical thicknesses are reduced to half those of a healthy CLN5^+/−^ sheep (Mitchell et al., 2023).

Unfortunately, due to limited vector availability, advanced symptomatic sheep could only be treated with a MD treatment and not the HD which had proved most efficacious in the early symptomatic cohort. Nevertheless, of the three CLN5^−/−^ sheep who received treatment at 9 months of age, one responded favorably (MD 1163–16). Disease progression was stabilized until 19 months post-treatment when the animal began to decline clinically, and it was euthanized at 30.9 months of age following increasing episodes of inducible tremors and stereotypical circling behavior. Intracranial volume loss was moderate, at 5.4 mL from 9 to 30.9 months, but significantly less than untreated CLN5^−/−^ sheep that lost an average of 9.4 mL between 3 and 19 months of age. In-life assessments of efficacy suggest that another MD ICV treated advanced symptomatic sheep (1143–16) was a non-responder, whilst the treatment had only minimal positive benefit to the third sheep. These two animals had fewer transduced cells, but neuropathological analyses also revealed amelioration of common disease markers (cortical thinning, astrocytosis, microgliosis, storage body accumulation) to a degree.

A number of gene therapy clinical trials have been performed, are underway, or are in preparation, for the different forms of NCL (https://clinicaltrials.gov/), including the first in-human CLN5 gene therapy study based on these and other efficacy studies in CLN5^−/−^ sheep (Clinicaltrials.gov identifier: NCT05228145). Routes of administration have typically been intraparenchymal (CLN2) or intrathecal into the lumbar cistern (CLN3, CLN6, CLN7). Pilot studies in a small number of healthy sheep indicate that the ICV route resulted in the most robust widespread distribution of vector throughout the entire CNS, compared to intracisternal magna, lumbar intrathecal or intraparenchymal delivery. Delivery directly into the cerebrospinal fluid (CSF) was preferred for CLN5 therapeutics, as it allows for maximum transduction of the CNS and circumvents the blood-brain and blood-CSF barriers, reducing the risk of an immune response or off-target side effects, seen with systemic (intravenous) delivery of AAV9 vectors and other biologics (Zincarelli et al., 2008; Xie et al., 2011; Sadekar et al., 2022). Increasingly the ICV delivery route is being recognized as a powerful way to transduce widespread areas of the brain in CNS disorders (Hughes et al., 2018; Bey et al., 2020; Hinderer et al., 2020; Galvan et al., 2021). It is the infusion route for the only current approved NCL enzyme replacement therapy, cerliponase alfa, for CLN2 disease (Schulz et al., 2018) and is the delivery route of choice to target the brain in the current CLN5 gene therapy clinical trial.

Consistent with similar studies in non-human primates (Naidoo et al., 2018; Bey et al., 2020; Galvan et al., 2021), this sheep study shows that the ICV delivery route did not result in high levels of transduction of deeper brain structures, such as the basal ganglia (striatum, putamen and caudate nucleus) or thalamus. However, failure to greatly transduce these regions that are not as severely affected in ovine CLN5 disease (Mitchell et al., 2023) did not result in increased subcortical disease pathology and seems unlikely to have contributed greatly to disease progression. Levels of neuroinflammation and storage burden in the thalamus and cerebellum of ICV treated sheep aged 17.9–60.1 months of age were not significantly different from much younger 24-month-old untreated CLN5^−/−^ sheep. In fact, the four early symptomatic treated sheep who received the highest ICV dose had significantly less thalamic gliosis and lysosomal storage accumulation in their thalamus and cerebellum than untreated CLN5^−/−^ sheep, suggesting higher doses might even attenuate subcortical pathology. Much discussion arises over the benefits of unilateral *versus* bilateral ICV delivery route. Whilst bilateral injections have been used in most pre-clinical animal studies, therapeutic benefit has been demonstrated in a canine model of CLN2 NCL after a single unilateral injection of AAV serotype 2 expressing the canine CLN2/TPP1 enzyme (Katz et al., 2015). Injections in this study were bilateral except for one (HD 1172-15) that received both doses into the right ventricle following difficulty in identifying the left ventricle. This had no apparent impact on the efficacy of the treatment. The sheep was clinically stable, lost minimal intracranial volume and was euthanized early at 22.3 months of age, whilst in excellent clinical health. Post-mortem neuropathological analysis revealed no significant difference in cortical thickness, neuroinflammatory marker expression, lysosomal storage burden or CLN5 expression between the two hemispheres suggesting widespread CNS transduction could be achieved by unilateral delivery. The only asymmetry was a larger amount of transduced sensory and motor neurons in the right column of the cervical spinal cord compared with the left column.

In line with previous NCL gene therapy studies in sheep and dogs (Katz et al., 2015; Mitchell et al., 2018). ICV delivery alone was not sufficient to protect against vision loss in the current study. Unilateral intravitreal (IVT) delivery of scAAV9/oCLN5 has resulted in maintenance of retinal structure and function in the treated eye of CLN5^−/−^ sheep for up to 15 months post-treatment, while the untreated eye displayed progressive electroretinographic decline and severe atrophy on post-mortem examination (Murray et al., 2021). A second IND-enabling study in sheep has subsequently shown that combined ocular and brain-directed gene therapy simultaneously halt neurological and retinal disease in ovine CLN5 Batten disease (Murray et al., 2023), prompting clinical translation of dual ICV and IVT administration for this disease.

Although the ICV delivery route is efficacious in the brain, one of the concerns with a non-systemic delivery route is that not all regions of the body are targeted. Typically, in NCL, pathology outside of the brain and eye have not been considered due to the profound effect of neurodegeneration, vision loss and death before other pathologies emerge. However, the *CLN5* gene mutation is present in every cell of the body, lysosomal storage is largely ubiquitous and in humans endogenous CLN5 protein is widely expressed (https://www.proteinatlas.org/). Extra-neuronal pathologies have previously been documented in the heart, liver and muscle tissue of CLN2^−/−^ dogs whose lives were significantly extended through ICV CLN2 gene therapy (Katz et al., 2017). Here, the HD treated sheep 1,164–15 died unexpectedly at 60.1 months of age from gastrointestinal stasis. Descriptions of constipation, abdominal pain and bloating, vomiting, gastric reflux, changes in gut microbiota, loss of enteric neurons and impaired gastrointestinal motility have been published and anecdotally reported for NCL patients and animal models (Mole et al., 2021; Parker et al., 2021; Wang et al., 2021) so long-term future therapeutic strategies may be required for other pathologies, such as those in the enteric nervous system.

There were other limitations to this study. Although histochemical analyses suggested the co-existence of vector-transduced cells and cross-corrected cells within the treated brain, it was not categorically possible to confirm that cross-correction was occurring in the ICV treated sheep brains. Strong CLN5-positive transduced cells were easily distinguished from diffuse weak CLN5-positive neighboring cells which were hypothesized to have taken up secreted CLN5 protein. However, as treated sheep brains were perfusion-fixed *in situ*, no fresh frozen brain tissue was available for vector copy number analyses for a direct comparison of CLN5 protein to AAV9 distribution.

The *CLN5* mutation in Borderdale sheep is caused by a splice site nucleotide substitution, resulting in the excision of exon 3 and a truncated protein (Frugier et al., 2008). Like sheep, many of the disease-causing mutations in humans result in a putative shorter CLN5 protein (Basak et al., 2021), however some patients will have mutations that preclude any CLN5 protein synthesis. Therefore, there is a risk that CLN5^−/−^ individuals, who lack expression of some or all natural CLN5 epitopes, could mount an immune response when they receive the *CLN5* transgene, however this does not appear to be an issue in sheep. Indeed, CNS and ocular gene therapy studies show that sheep appear to be tolerant to native and non-native transgenes (Banin et al., 2015; Gray-Edwards et al., 2018; O’Leary et al., 2023). In the current study, there was no apparent upregulation in neuroinflammation in treated sheep that could not be considered disease-associated, and there was long-term transgene expression and no other clinical indications of toxicity. However, as the sheep did not receive any immunosuppressive drugs, this study did not provide translational data on the need for immunomodulation in human CLN5 patients.

Finally, the ovine and human *CLN5* transgenes used in this study were codon optimized. There is concern that this is not necessary for expression of a gene in its native host and the process itself might affect protein conformation and function, detrimentally increasing immunogenicity or reducing efficacy (Mauro and Chappell, 2014). Here, codon optimization was performed to strengthen the expression cassette, ensure robust protein expression, and minimize the potential for cryptic splice sites and start codons which might produce non-specific or immunogenic peptides. Whilst the native sequence is preferred, it was evident from this study that codon optimization did not adversely affect functionality and the first-in-human CLN5 gene therapy Phase I/II Clinical Trial (NCT05228145) supported by these sheep studies will also use a codon-optimised human *CLN5* transgene.

Overall, these studies in CLN5^−/−^ sheep demonstrate the long-term efficacy of ICV scAAV9/oCLN5 gene therapy when delivered at pre-symptomatic, early symptomatic, and more advanced symptomatic disease stages at the doses administered. Treatment was well tolerated, delayed disease progression, stereotypical brain atrophy, and disease pathology in the majority of the treated sheep but did not preserve vision at any dose or disease state evaluated. In preparation for clinical translation, *in vitro* studies indicated higher expression levels of the proposed investigational drug, AAV9 expressing the human *CLN5* gene, than the ovine gene therapy product delivered *in vivo*. Typically for lysosomal storage diseases like CLN5 NCL, the earlier the treatment the better (Sands and Davidson, 2006), yet the findings from early and advanced symptomatic treated sheep demonstrate dose-dependent transduction efficiency is important. Here, the MD and HD treatments were the most efficacious, prompting further studies in sheep with even higher doses and the inclusion of an ocular delivery route for retinal targeting (Murray et al., 2023). This study indicates great translational promise for extending and improving the quality of life in post-symptomatic CLN5 patients, so long as they have sufficient remaining cortical and cerebellar neurons to benefit from CLN5 expression and that higher doses are likely to be more effective.

## Data Availability

The raw data supporting the conclusion of this article will be made available by the authors, without undue reservation.

## References

[B1] Banin E. Gootwine E. Obolensky A. Ezra-Elia R. Ejzenberg A. Zelinger L. (2015). Gene augmentation therapy restores retinal function and visual behavior in a sheep model of CNGA3 achromatopsia. Mol. Ther. 23, 1423–1433. 10.1038/mt.2015.114 26087757 PMC4817879

[B2] Basak I. Wicky H. E. McDonald K. O. Xu J. B. Palmer J. E. Best H. L. (2021). A lysosomal enigma CLN5 and its significance in understanding neuronal ceroid lipofuscinosis. Cell. Mol. Life Sci. 78, 4735–4763. 10.1007/s00018-021-03813-x 33792748 PMC8195759

[B3] Bey K. Deniaud J. Dubreil L. Joussemet B. Cristini J. Ciron C. (2020). Intra-CSF AAV9 and AAVrh10 administration in nonhuman primates: Promising routes and vectors for which neurological diseases? Mol. Ther. - Methods Clin. Dev. 17, 771–784. 10.1016/j.omtm.2020.04.001 32355866 PMC7184633

[B4] Federov A. Beichel R. Kalpathy-Cramer J. Finet J. Fillion-Robin J.-C. Pujol S. (2012). 3D Slicer as an image computing platform for the quantitative imaging network. Magn. Reson. Imaging 30, 1323–1341. 10.1016/j.mri.2012.05.001 22770690 PMC3466397

[B5] Frugier T. Mitchell N. L. Tammen I. Houweling P. J. Arthur D. G. Kay G. W. (2008). A new large animal model of CLN5 neuronal ceroid lipofuscinosis in Borderdale sheep is caused by a nucleotide substitution at a consensus splice site (c.571 + 1G > A) leading to excision of exon 3. Neurobiol. Dis. 29, 306–315. 10.1016/j.nbd.2007.09.006 17988881 PMC2249613

[B6] Galvan A. Petkau T. L. Hill A. M. Korecki A. J. Lu G. Choi Di. (2021). Intracerebroventricular administration of AAV9-PHP.B SYN1-EmGFP induces widespread transgene expression in the mouse and monkey central nervous system. Hum. Gene Ther. 32, 599–615. 10.1089/hum.2020.301 33860682 PMC8236560

[B7] Gray-Edwards H. L. Randle A. N. Maitland S. A. Benatti H. R. Hubbard S. M. Canning P. F. (2018). Adeno-associated virus gene therapy in a sheep model of Tay-Sachs disease. Hum. Gene Ther. 29, 312–326. 10.1089/hum.2017.163 28922945

[B8] Grieger J. C. Soltys S. M. Samulski R. J. (2016). Production of recombinant adeno-associated virus vectors using suspension HEK293 cells and continuous harvest of vector from the culture media for GMP FIX and FLT1 clinical vector. Mol. Ther. 24, 287–297. 10.1038/mt.2015.187 26437810 PMC4817810

[B9] Hinderer C. Nosratbakhsh B. Katz N. Wilson J. M. (2020). A single injection of an optimized adeno-associated viral vector into cerebrospinal fluid corrects neurological disease in a murine model of GM1 gangliosidosis. Hum. Gene Ther. 31, 1169–1177. 10.1089/hum.2018.206 33045869 PMC7698982

[B10] Hughes M. P. Smith D. A. Morris L. Fletcher C. Colaco A. Huebecker M. (2018). AAV9 intracerebroventricular gene therapy improves lifespan, locomotor function and pathology in a mouse model of Niemann-Pick type C1 disease. Hum. Mol. Genet. 27, 3079–3098. 10.1093/hmg/ddy212 29878115 PMC6097154

[B11] Isosomppi J. Vesa J. Jalanko A. Peltonen L. (2002). Lysosomal localization of the neuronal ceroid lipofuscinosis CLN5 protein. Hum. Mol. Genet. 11, 885–891. 10.1093/hmg/11.8.885 11971870

[B12] Jolly R. D. Arthur D. G. Kay G. W. Palmer D. N. (2002). Neuronal ceroid-lipofuscinosis in Borderdale sheep. N. Z. Vet. J. 50, 199–202. 10.1080/00480169.2002.36311 16032271

[B13] Katz M. L. Johnson G. C. Leach S. B. Williamson B. G. Coates J. R. Whiting R. E. H. (2017). Extraneuronal pathology in a canine model of CLN2 neuronal ceroid lipofuscinosis after intracerebroventricular gene therapy that delays neurological disease progression. Gene Ther. 24, 215–223. 10.1038/gt.2017.4 28079862 PMC5398942

[B14] Katz M. L. Tecedor L. Chen Y. Williamson B. G. Lysenko E. Wininger F. A. (2015). AAV gene transfer delays disease onset in a TPP1-deficient canine model of the late infantile form of Batten disease. Sci. Transl. Med. 7, 313ra180. 10.1126/scitranslmed.aac6191 PMC4968409 26560358

[B15] Lévy M. (2020). Polioencephalomalacia in ruminants (Cerebrocortical necrosis). Merck Sharpe Dohme Corp. MSD Vet. Man. Available at: https://www.msdvetmanual.com/nervous-system/polioencephalomalacia/polioencephalomalacia-in-ruminants .

[B16] Linterman K. S. Palmer D. N. Kay G. W. Barry L. A. Mitchell N. L. McFarlane R. G. (2011). Lentiviral-mediated gene transfer to the sheep brain: Implications for gene therapy in batten disease. Hum. Gene Ther. 22, 1011–1020. 10.1089/hum.2011.026 21595499 PMC3159522

[B17] Mauro V. P. Chappell S. A. (2014). A critical analysis of codon optimization in human therapeutics. Trends Mol. Med. 20, 604–613. 10.1016/j.molmed.2014.09.003 25263172 PMC4253638

[B18] Mitchell N. L. Russell K. N. Barrell G. K. Tammen I. Palmer D. N. (2023). Characterization of neuropathology in ovine CLN5 and CLN6 neuronal ceroid lipofuscinoses (Batten disease). Dev. Neurobiol. 10.1002/dneu.22918 37246363

[B19] Mitchell N. L. Russell K. N. Wellby M. P. Wicky H. E. Schoderboeck L. Barrell G. K. (2018). Longitudinal *in vivo* monitoring of the CNS demonstrates the efficacy of gene therapy in a sheep model of CLN5 batten disease. Mol. Ther. 26, 2366–2378. 10.1016/j.ymthe.2018.07.015 30078766 PMC6171082

[B20] Mole S. E. Schulz A. Badoe E. Berkovic S. F. de Los Reyes E. C. Dulz S. (2021). Guidelines on the diagnosis, clinical assessments, treatment and management for CLN2 disease patients. Orphanet J. Rare Dis. 16, 185–219. 10.1186/s13023-021-01813-5 33882967 PMC8059011

[B21] Mole S. E. , Williams R. E. (2013). Neuronal ceroid lipofuscinoses. GeneReviews. Available at: https://www.ncbi.nlm.nih.gov/books/NBK1428/.

[B22] Murray S. J. Russell K. N. Melzer T. R. Gray S. J. Heap S. J. Palmer D. N. (2021). Intravitreal gene therapy protects against retinal dysfunction and degeneration in sheep with CLN5 Batten disease. Exp. Eye Res. 207, 108600. 10.1016/j.exer.2021.108600 33930398

[B23] Murray S. J. Wellby M. P. Barrell G. K. Russell K. N. Deane A. R. Wynyard J. R. (2023). Efficacy of dual intracerebroventricular and intravitreal CLN5 gene therapy in sheep prompts the first clinical trial to treat CLN5 Batten disease. Front. Pharmacol [pre-print]. 10.3389/fphar.2023.1212235 PMC10628725 37942487

[B24] Naidoo J. Stanek L. M. Ohno K. Trewman S. Samaranch L. Hadaczek P. (2018). Extensive transduction and enhanced spread of a modified AAV2 capsid in the non-human primate CNS. Mol. Ther. 26, 2418–2430. 10.1016/j.ymthe.2018.07.008 30057240 PMC6171051

[B25] Nita D. A. Mole S. E. Minassian B. A. (2016). Neuronal ceroid lipofuscinoses. Epileptic Disord. 18, 73–88. 10.1684/epd.2016.0844 27629553

[B26] O’Leary C. Forte G. Mitchell N. L. Youshani A. S. Dyer A. Wellby M. P. (2023). Intraparenchymal convection enhanced delivery of AAV in sheep to treat Mucopolysaccharidosis IIIC. J. Transl. Med. 21, 437. 10.1186/s12967-023-04208-1 37407981 PMC10320977

[B27] Oswald M. J. Palmer D. N. Kay G. W. Shemilt S. J. A. Rezaie P. Cooper J. D. (2005). Glial activation spreads from specific cerebral foci and precedes neurodegeneration in presymptomatic ovine neuronal ceroid lipofuscinosis (CLN6). Neurobiol. Dis. 20, 49–63. 10.1016/j.nbd.2005.01.025 16137566

[B28] Parker C. Zhao J. Pearce D. A. Kovács A. D. (2021). Comparative analysis of the gut microbiota composition in the Cln1 R151X and Cln2 R207X mouse models of Batten disease and in three wild-type mouse strains. Arch. Microbiol. 203, 85–96. 10.1007/s00203-020-02007-6 32749661

[B29] Russell K. N. Mitchell N. L. Anderson N. G. Bunt C. R. Wellby M. P. Melzer T. R. (2018). Computed tomography provides enhanced techniques for longitudinal monitoring of progressive intracranial volume loss associated with regional neurodegeneration in ovine neuronal ceroid lipofuscinoses. Brain Behav. 8, e01096. 10.1002/brb3.1096 30136763 PMC6160654

[B30] Russell K. N. Mitchell N. L. Wellby M. P. Barrell G. K. Palmer D. N. (2021). Electroretinography data from ovine models of CLN5 and CLN6 neuronal ceroid lipofuscinoses. Data Br. 37, 107188. 10.1016/j.dib.2021.107188 PMC8187955 34141843

[B31] Sadekar S. S. Bowen M. Cai H. Jamalian S. Rafidi H. Shatz‐Binder W. (2022). Translational approaches for brain delivery of biologics via cerebrospinal fluid. Clin. Pharmacol. Ther. 111, 826–834. 10.1002/cpt.2531 35064573 PMC9305158

[B32] Sands M. S. Davidson B. L. (2006). Gene therapy for lysosomal storage diseases. Mol. Ther. 13, 839–849. 10.1016/j.ymthe.2006.01.006 16545619

[B33] Schulz A. Ajayi T. Specchio N. de Los Reyes E. Gissen P. Ballon D. (2018). Study of intraventricular cerliponase alfa for CLN2 disease. N. Engl. J. Med. 378, 1898–1907. 10.1056/NEJMoa1712649 29688815

[B34] Simonati A. Williams R. E. Nardocci N. Laine M. Battini R. Schulz A. (2017). Phenotype and natural history of variant late infantile ceroid-lipofuscinosis 5. Dev. Med. Child. Neurol. 59, 815–821. 10.1111/dmcn.13473 28542837

[B35] Wang S. H. Williams E. M. Takahashi K. Nelvagal H. R. B A. J. Makita T. (2021). “Enteric nervous system defects underlie bowel dysfunction in Cln1, Cln2 and Cln3 disease mice: A new therapeutic target?,” in Proccedings of the 17th International Conference on Neuronal Ceroid Lipofuscinoses (Batten Disease), St Louis, MO, USA, September 2021.

[B36] Xie J. Xie Q. Zhang H. Ameres S. L. Hung J.-H. Su Q. (2011). MicroRNA-regulated, systemically delivered rAAV9: A step closer to CNS-restricted transgene expression. Mol. Ther. 19, 526–535. 10.1038/mt.2010.279 21179009 PMC3048189

[B37] Xin W. Mullen T. E. Kiely R. Min J. Feng X. Cao Y. (2010). CLN5 mutations are frequent in juvenile and late-onset non-Finnish patients with NCL. Neurology 74, 565–571. 10.1212/WNL.0b013e3181cff70d 20157158

[B38] Zincarelli C. Soltys S. Rengo G. Rabinowitz J. E. (2008). Analysis of AAV serotypes 1-9 mediated gene expression and tropism in mice after systemic injection. Mol. Ther. 16, 1073–1080. 10.1038/mt.2008.76 18414476

