# Supplementary material for: Long-term safety and dose escalation of intracerebroventricular CLN5 gene therapy in sheep supports clinical translation for CLN5 Batten disease
Source: Front Genet. 2023 Aug 8;14:1212228. doi: 10.3389/fgene.2023.1212228 (PMC10442658; doi:10.3389/fgene.2023.1212228)
Supplement: Supplementary file 1 [file DataSheet1.pdf]

Supplementary Material

Long-term safety and dose escalation of intracerebroventricular CLN5 gene therapy in sheep supports clinical translation for CLN5 Batten disease.

Nadia L. Mitchell*, Samantha J. Murray, Martin P. Wellby, Graham K. Barrell, Katharina N. Russell, Ashley R. Deane, John R. Wynyard, Madeleine J. Palmer, Anila Pulickan, Phillipa M. Prendergast, Widler Casy, Steven J. Gray, David N. Palmer

* **Correspondence:** Nadia Mitchell: Nadia.Mitchell@lincoln.ac.nz

Supplementary Table 1. Intracerebroventricular CLN5 gene therapy study design.

Study	Vector and transgene	Treatment	Dose[a]	Total dose (vg)	Volume (µl)	Sheep *n*	Genetic status	Treatment age (months)	Viral titer (vg/mL)
Pre-symptomatic	scAAV9/oCLN5	Bilateral ICV	LD/1	8.0×10^{10}	800	3	$CLN5^{-/-}$	2.9-3.2	1.6×10^{12}
			LD/2	8.2×10^{10}	800	3	$CLN5^{-/-}$	3.0-3.5	1.4×10^{13}
			MD	2.4×10^{11}	800	3	$CLN5^{-/-}$	2.9-3.2	1.6×10^{12}
Early symptomatic	scAAV9/oCLN5	Bilateral ICV	MD	2.7×10^{11}	800	3	$CLN5^{-/-}$	6.0	1.6×10^{12}
			HD	2.8×10^{12}	800	4	$CLN5^{-/-}$	7.3-7.5	4.9×10^{12}
Advanced symptomatic	scAAV9/oCLN5	Bilateral ICV	MD	2.7×10^{11}	800	3	$CLN5^{-/-}$	8.8-9.0	1.6×10^{12}
Control	N/A	Nil	N/A	N/A	N/A	14	$CLN5^{+/-}$	N/A	N/A
Affected	N/A	Nil	N/A	N/A	N/A	15	$CLN5^{-/-}$	N/A	N/A

[a] The LD treatments were delivered over two different years (LD/1 and LD/2)

Abbreviations: HD high dose; ICV intracerebroventricular; LD low dose; m months; MD moderate dose; n number, N/A not applicable; vg viral genomes

Supplementary Table 2. Cortical thickness following ICV treatment with scAAV9/oCLN5. Mean cortical thickness measurements (± SEM) for CLN5$^{-/-}$ sheep treated intracerebroventricularly with scAAV9/oCLN5 at 3 (pre-symptomatic), 6 (early symptomatic), or 9 (advanced-symptomatic) months of age were compared with healthy control CLN5$^{+/-}$ and untreated CLN5$^{-/-}$ sheep. At least 25 thickness measurements were taken per hemisphere per animal through three different cortical regions and the cerebellum on Nissl-stained sections.

Treatment	Sheep	Age at death (m)	Visual cortex		Parieto-occipital cortex		Motor cortex		Cerebellum	
			Thickness (µm)	SEM	Thickness (µm)	SEM	Thickness (µm)	SEM	Thickness (µm)	SEM
Healthy control CLN5$^{+/-}$	1103C	24.0	1893	334	2023	299	2356	198	506	39
	1111C	24.1	1904	229	1912	127	2376	266	510	62
	39C	24.2	2097	249	2006	133	2394	413	501	42
Untreated CLN5$^{-/-}$	1109U	23.2	908	141	1007	100	1094	174	507	67
	1104U	23.7	788	127	845	103	1095	80	502	70
	1116U	23.4	837	118	887	105	1395	314	496	120
Pre-symptomatic Low dose	1102-16	53.8	889	215	1384	93	1866	283	497	107
	1110-18	30.4	1173	167	1314	227	1761	172	495	92
	1111-16	47.3	753	102	826	137	1377	318	486	89
	1115-18	17.9	1442	147	1328	140	1445	148	499	112
	1122-18	24.0	1049	173	1171	153	1660	140	489	95
	1128-16	53.5	871	142	1109	178	1372	153	489	81
Pre-symptomatic Moderate dose	1104-16	27.4	1380	182	1594	232	1869	425	496	94
	1120-16	50.4	1003	147	1317	168	1538	122	491	58
	1123-16	44.7	830	191	1332	170	1520	113	480	82
Early symptomatic Moderate dose	1185-16	21.4	1233	167	1181	128	1943	178	499	93
	1186-16	49.6	986	154	1003	122	1520	306	503	131
	1187-16	49.6	819	92	1039	121	1411	216	491	114
Early symptomatic High dose	1164-15	60.1	1186	191	1186	191	1579	363	498	107
	1165-15	22.5	1219	140	1430	127	1666	237	502	109
	1170-15	42.3	1054	189	1213	213	1662	303	496	99
	1172-15	22.3	1350	273	1436	98	1832	233	497	119
Advanced symptomatic Moderate dose	1143-16	18.5	984	176	1055	116	1706	155	494	100
	1163-16	30.9	1091	119	1186	83	1908	214	504	82
	1165-16	21.6	817	87	1156	150	1719	283	496	69

Abbreviations: m months

Supplementary Table 3. Astrocytic response following ICV treatment with scAAV9/oCLN5. Mean percent area of GFAP immunostaining (± SEM) for $CLN5^{-/-}$ sheep treated intracerebroventricularly with scAAV9/oCLN5 at 3 (pre-symptomatic), 6 (early symptomatic), or 9 (advanced-symptomatic) months of age were compared with healthy control $CLN5^{+/-}$ and untreated $CLN5^{-/-}$ sheep. At least 10 percentage area measurements were taken per hemisphere per animal through five different brain regions on GFAP-immunostained sections.

Treatment	Sheep	Age at death (m)	Visual cortex		Parieto-occipital cortex		Motor cortex		Thalamus		Cerebellum	
			% area	SEM	% area	SEM	% area	SEM	% area	SEM	% area	SEM
Healthy control $CLN5^{+/-}$	295C	24.0	2.5	0.2	2.1	0.4	2.1	1.5	2.0	0.4	2.5	0.1
	1111C	24.1	2.6	1.3	2.2	0.9	2.1	0.7	1.5	0.2	2.3	0.2
	39C	24.2	2.2	0.7	2.5	0.9	1.8	0.7	1.9	0.1	2.4	0.2
Untreated $CLN5^{-/-}$	1109U	23.2	16.8	2.0	24.8	2.5	16.7	3.1	3.9	0.4	2.6	0.1
	1104U	23.7	16.6	4.5	19.8	3.3	16.6	3.8	4.1	0.5	2.5	0.1
	1119U	23.4	16.6	3.8	21.8	2.0	15.8	2.7	4.0	0.7	2.7	0.1
Pre-symptomatic Low dose	1102-16	53.8	17.5	3.9	15.5	4.7	4.4	1.4	2.9	0.5	2.5	0.4
	1110-18	30.4	14.3	1.2	21.6	2.0	8.6	2.2	4.2	0.5	2.7	0.2
	1111-16	47.3	9.3	2.1	25.9	2.1	2.6	1.3	4.4	0.3	2.9	0.2
	1115-18	17.9	7.8	3.5	11.4	6.0	2.5	2.6	3.3	0.4	2.4	0.2
	1122-18	24.0	6.8	3.1	14.3	2.7	5.1	2.4	2.9	0.3	2.5	0.6
	1128-16	53.5	3.9	1.0	12.7	5.6	3.9	3.5	4.1	0.2	2.5	0.5
Pre-symptomatic Moderate dose	1104-16	27.4	6.8	2.0	19.9	5.3	3.2	1.3	2.0	0.3	2.3	0.5
	1120-16	50.4	4.0	0.9	5.7	1.8	3.7	3.1	4.4	0.4	2.6	0.2
	1123-16	44.7	6.8	2.0	16.9	9.1	2.8	1.3	3.3	0.4	2.6	0.5
Early symptomatic Moderate dose	1185-16	21.4	6.9	2.4	9.8	5.0	2.2	0.7	2.0	0.3	2.4	0.3
	1186-16	49.6	7.7	2.7	9.9	5.9	3.8	1.7	3.7	0.4	2.6	0.3
	1187-16	49.6	10.6	2.2	13.1	2.3	5.0	1.8	3.9	0.4	2.7	0.3
Early symptomatic High dose	1164-15	60.1	9.2	1.2	18.0	1.7	5.9	1.8	2.5	0.2	2.5	0.3
	1165-15	22.5	12.9	3.0	14.7	2.3	7.8	2.6	2.5	0.3	2.4	0.4
	1170-15	42.3	8.1	3.2	8.4	2.9	1.9	0.9	2.8	0.1	2.3	0.3
	1172-15	22.3	12.6	1.5	15.0	2.7	8.1	3.5	2.6	0.3	2.5	0.3

Advanced symptomatic Moderate dose	1143-16	18.5	16.1	2.8	15.4	2.0	5.7	5.3	4.3	0.3	2.5	0.4
	1163-16	30.9	12.6	2.8	13.1	2.4	4.2	1.4	2.4	0.2	2.4	0.4
	1165-16	21.6	17.8	2.8	15.6	3.3	9.5	2.4	2.5	0.2	2.5	0.3

Abbreviations: m months

Supplementary Table 4. Microglial response following ICV treatment with scAAV9/oCLN5. Mean percent area of GSB4 staining (± SEM) for CLN5$^{-/-}$ sheep treated intracerebroventricularly with scAAV9/oCLN5 at 3 (pre-symptomatic), 6 (early symptomatic), or 9 (advanced symptomatic) months of age were compared with healthy control CLN5$^{+/-}$ and untreated CLN5$^{-/-}$ sheep. At least 10 percentage area measurements were taken per hemisphere per animal through five different brain regions on GSB4-stained sections.

Treatment	Sheep	Age at death (m)	Visual cortex		Parieto-occipital cortex		Motor cortex		Thalamus		Cerebellum	
			% area	SEM	% area	SEM	% area	SEM	% area	SEM	% area	SEM
Healthy control CLN5$^{+/-}$	295C	24.0	0.4	0.4	0.5	0.2	0.7	0.2	0.2	0.0	3.1	0.1
	1111C	24.1	0.5	0.3	0.8	0.6	0.4	0.3	0.2	0.1	3.0	0.2
	39C	24.2	0.3	0.1	0.6	0.2	0.5	0.3	0.2	0.1	2.8	0.1
Untreated CLN5$^{-/-}$	1109U	23.2	6.9	0.4	7.5	1.1	4.5	1.3	1.9	0.2	2.9	0.2
	1104U	23.7	7.3	1.7	7.5	1.5	4.5	1.4	1.3	0.2	3.0	0.2
	1119U	23.4	5.7	1.5	5.2	1.0	3.4	1.3	2.0	0.2	3.1	0.2
Pre-symptomatic Low dose	1102-16	53.8	7.2	2.2	3.6	2.3	4.0	1.8	2.1	0.3	2.9	0.6
	1110-18	30.4	6.6	4.7	6.6	5.5	4.7	1.2	1.5	0.3	3.1	0.4
	1111-16	47.3	3.0	1.9	3.8	1.8	0.3	0.2	2.0	0.3	2.9	0.3
	1115-18	17.9	3.2	1.1	2.6	1.0	0.6	0.5	1,6	0.4	3.1	0.3
	1122-18	24.0	3.2	0.9	2.5	1.4	2.7	1.2	1.3	0.4	2.9	0.3
	1128-16	53.5	2.6	0.7	3.7	1.2	1.5	0.9	0.6	0.3	2.9	0.4
Pre-symptomatic Moderate dose	1104-16	27.4	1.9	0.6	1.6	0.6	3.6	1.4	0.2	0.1	2.6	0.3
	1120-16	50.4	3.8	0.8	6.9	3.7	3.9	1.8	0.4	0.2	3.0	0.3
	1123-16	44.7	7.2	2.7	7.7	2.5	3.5	2.2	0.6	0.4	2.7	0.4
Early symptomatic Moderate dose	1185-16	21.4	5.1	0.7	4.9	1.6	1.5	0.4	1.2	0.3	3.1	0.4
	1186-16	49.6	3.1	3.1	2.5	1.1	1.8	1.9	0.9	0.6	3.0	0.3
	1187-16	49.6	2.1	1.2	1.4	0.6	2.4	2.1	1.7	0.5	3.0	0.3
Early symptomatic High dose	1164-15	60.1	4.0	0.9	4.0	1.8	3.2	1.7	1.0	0.2	3.1	0.5
	1165-15	22.5	5.0	1.3	4.8	0.9	1.9	0.6	1.3	0.5	2.9	0.4
	1170-15	42.3	4.0	2.8	3.7	1.1	0.9	0.9	1.4	0.8	3.1	0.3
	1172-15	22.3	4.9	1.5	4.3	1.1	1.8	0.6	0.6	0.5	3.0	0.2

Advanced symptomatic Moderate dose	1143-16	18.5	5.2	1.3	7.3	0.9	2.0	0.7	1.7	0.3	3.2	0.3
	1163-16	30.9	2.8	0.7	2.9	0.7	1.4	0.5	1.0	0.6	3.0	0.3
	1165-16	21.6	6.4	2.3	4.5	1.4	1.8	0.5	1.3	0.3	2.6	0.3

Abbreviations: m months

Supplementary Table 5. Lysosomal storage following ICV treatment with scAAV9/oCLN5. Mean percent area of fluorescence (± SEM) for $CLN5^{-/-}$ sheep treated intracerebroventricularly with scAAV9/oCLN5 at 3 (pre-symptomatic), 6 (early symptomatic), or 9 (advanced-symptomatic) months of age were compared with healthy control $CLN5^{+/-}$ and untreated $CLN5^{-/-}$ sheep. At least 10 percentage area measurements were taken per hemisphere per animal through five different brain regions on unstained sections.

Treatment	Sheep	Age at death (m)	Visual cortex		Parieto-occipital cortex		Motor cortex		Thalamus		Cerebellum	
			% area	SEM	% area	SEM	% area	SEM	% area	SEM	% area	SEM
Healthy control $CLN5^{+/-}$	295C	24.0	0.3	0.2	0.0	0.1	0.1	0.2	0.0	0.0	0.0	0.0
	1111C	24.1	0.0	0.0	0.0	0.0	0.0	0.0	0.1	0.1	0.0	0.0
	39C	24.2	0.2	0.3	0.2	0.2	0.1	0.3	0.1	0.1	0.0	0.0
Untreated $CLN5^{-/-}$	1109U	23.2	6.3	1.1	6.2	1.3	4.6	1.8	4.5	0.7	1.6	0.2
	1104U	23.7	6.5	1.5	6.4	1.4	5.3	0.8	4.4	0.5	1.6	0.4
	1119U	23.4	6.1	1.4	6.5	0.9	4.4	1.8	4.3	0.5	1.4	0.2
Pre-symptomatic Low dose	1102-16	53.8	6.5	1.0	6.4	1.7	5.0	1.5	2.9	0.5	1.4	0.6
	1110-18	30.4	6.3	1.4	6.7	1.8	4.6	2.1	3.8	0.2	0.8	0.4
	1111-16	47.3	6.0	1.5	6.2	1.5	5.4	1.4	3.5	0.3	1.5	0.4
	1115-18	17.9	5.8	1.1	5.7	1.0	4.0	1.1	3.9	0.4	1.4	0.3
	1122-18	24.0	3.8	1.5	4.1	2.1	2.3	1.0	4.1	0.4	0.7	0.2
	1128-16	53.5	5.9	0.9	5.8	1.2	4.1	0.8	3.8	0.5	0.8	0.3
Pre-symptomatic Moderate dose	1104-16	27.4	2.5	0.2	4.1	1.0	1.4	0.4	1.6	0.3	1.5	0.3
	1120-16	50.4	6.0	1.5	5.4	0.7	3.0	0.8	1.6	0.3	1.4	0.3
	1123-16	44.7	7.0	2.5	6.4	2.1	5.4	1.4	3.7	0.4	1.7	0.3
Early symptomatic Moderate dose	1185-16	21.4	3.3	0.8	4.9	0.9	2.4	0.2	1.8	0.2	1.5	0.2
	1186-16	49.6	3.6	0.7	4.6	0.8	2.5	0.5	4.2	0.3	0.9	0.3
	1187-16	49.6	3.0	0.9	3.8	0.5	2.3	0.5	3.2	0.4	1.3	0.3
Early symptomatic High dose	1164-15	60.1	3.8	2.0	4.1	0.9	4.3	1.5	1.9	0.7	1.1	0.2
	1165-15	22.5	4.8	1.0	4.9	0.9	2.6	0.4	2.1	0.4	0.7	0.3
	1170-15	42.3	4.3	1.0	4.4	0.6	2.6	0.7	1.7	0.3	0.8	0.2
	1172-15	22.3	4.8	0.9	5.0	1.0	2.4	0.5	1.7	0.4	0.5	0.1

Advanced symptomatic Moderate dose	1143-16	18.5	5.9	1.4	6.5	0.3	3.0	0.8	4.3	0.2	1.5	0.3
	1163-16	30.9	3.4	0.5	5.1	0.7	2.2	0.4	1.2	0.3	0.7	0.4
	1165-16	21.6	5.8	0.5	5.9	0.3	4.1	0.6	2.9	0.5	1.3	0.2

Abbreviations: m months

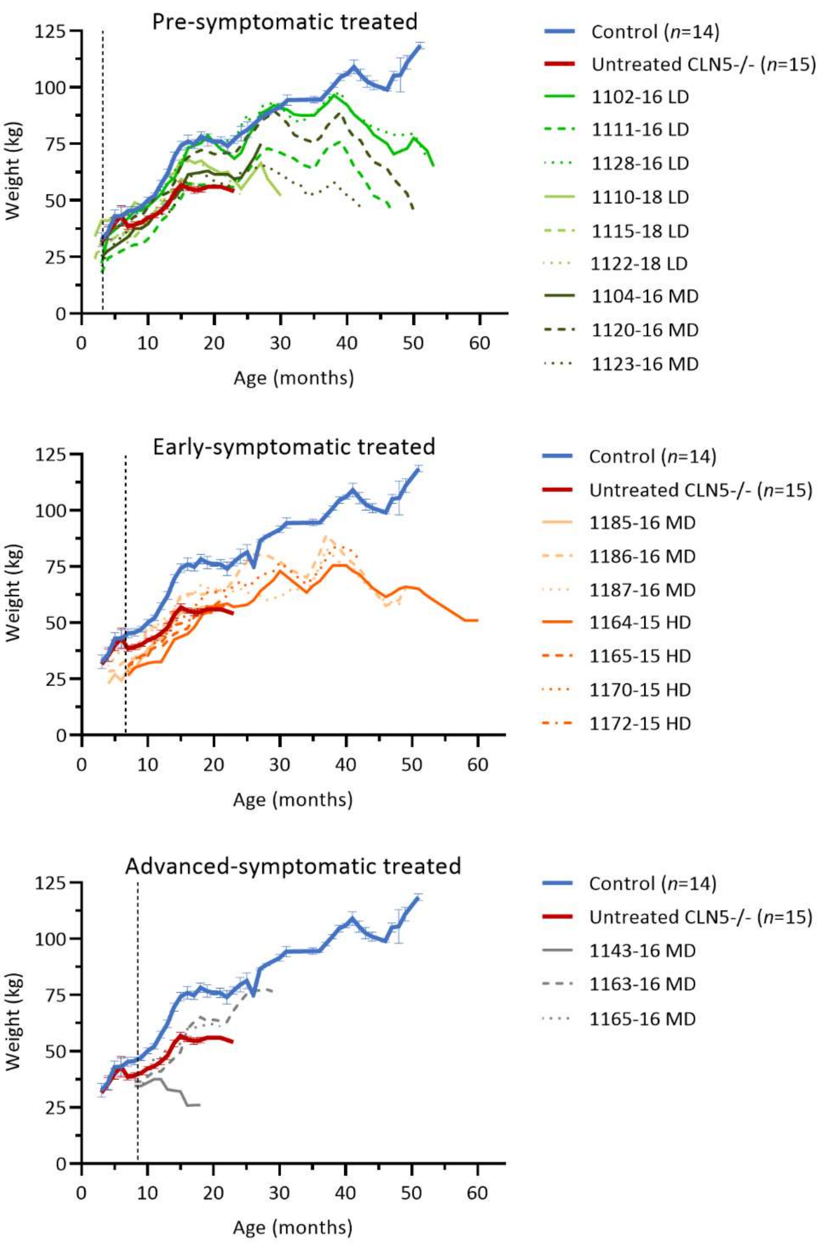

Supplementary Figure 1. Healthy weight gains were reported for most sheep after ICV scAAV9/oCLN5. Individual weight changes in ICV treated $CLN5^{-/-}$ sheep were compared with average data from healthy control $CLN5^{+/-}$ (blue) and untreated $CLN5^{-/-}$ (red) sheep. Many of the treated sheep reached healthy adult weights over their lifetime, but lost weight prior to euthanasia. Weights are displayed by treatment group (pre-symptomatic, green; early symptomatic, orange; advanced symptomatic, grey) at low (LD), moderate (MD) or high (HD) doses. Control data (*n*=2-4) was collected concurrently with each study and pooled for presentation. Dashed lines indicate treatment age.

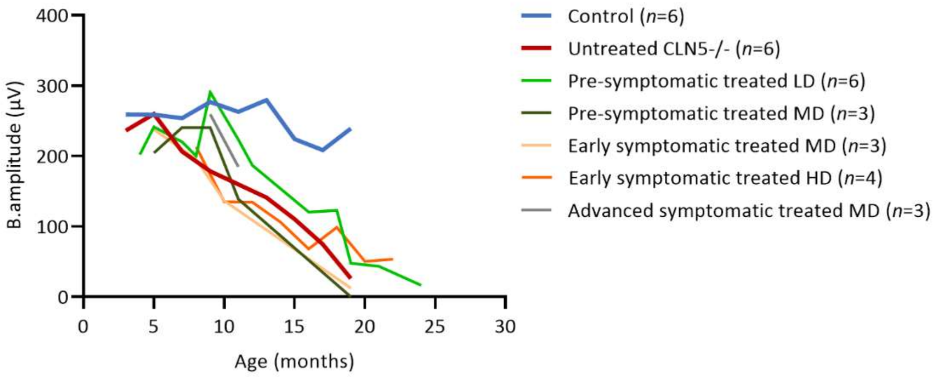

Supplementary Figure 2. ICV scAAV9/oCLN5 does not protect vision. Average ERG B-wave amplitude changes in ICV treated $CLN5^{-/-}$ sheep were compared with average historic data from healthy control $CLN5^{+/-}$ (blue) and untreated $CLN5^{-/-}$ (red) sheep. These show that the ICV treatment alone was not sufficient to protect vision long-term. Treatment ages were pre-symptomatic (green), early symptomatic (orange) or advanced symptomatic (grey) whilst doses were low (LD), moderate (MD) or high dose (HD).

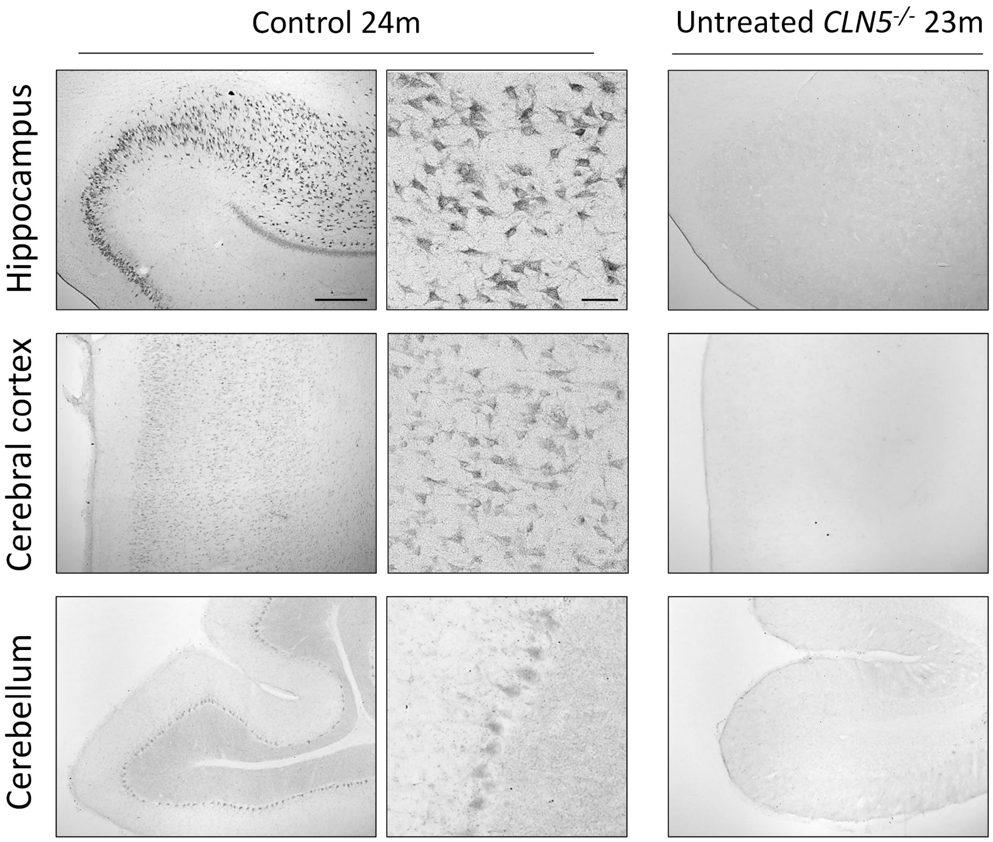
Control 24m
Untreated CLN5-/- 23m
Hippocampus
Cerebral cortex
Cerebellum

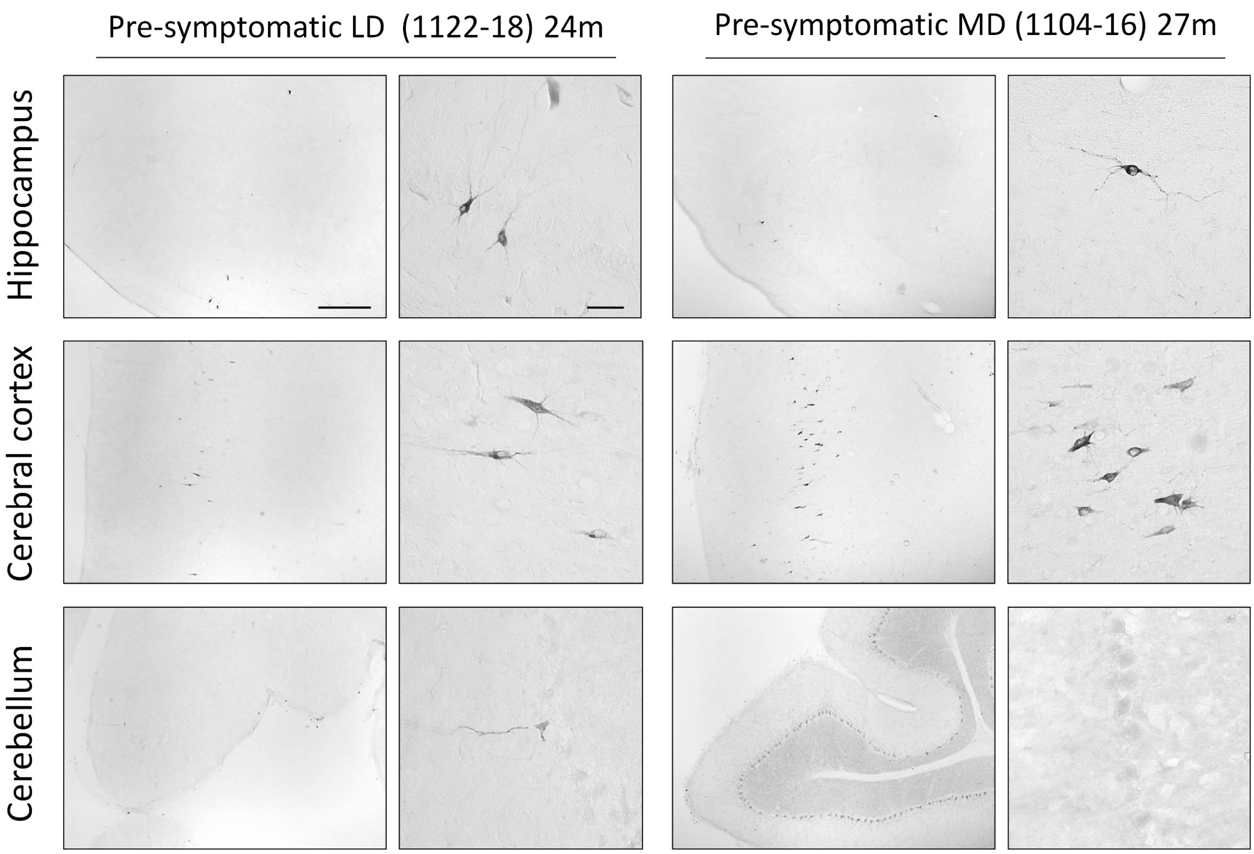
Pre-symptomatic LD (1122-18) 24m
Pre-symptomatic MD (1104-16) 27m
Hippocampus
Cerebral cortex
Cerebellum

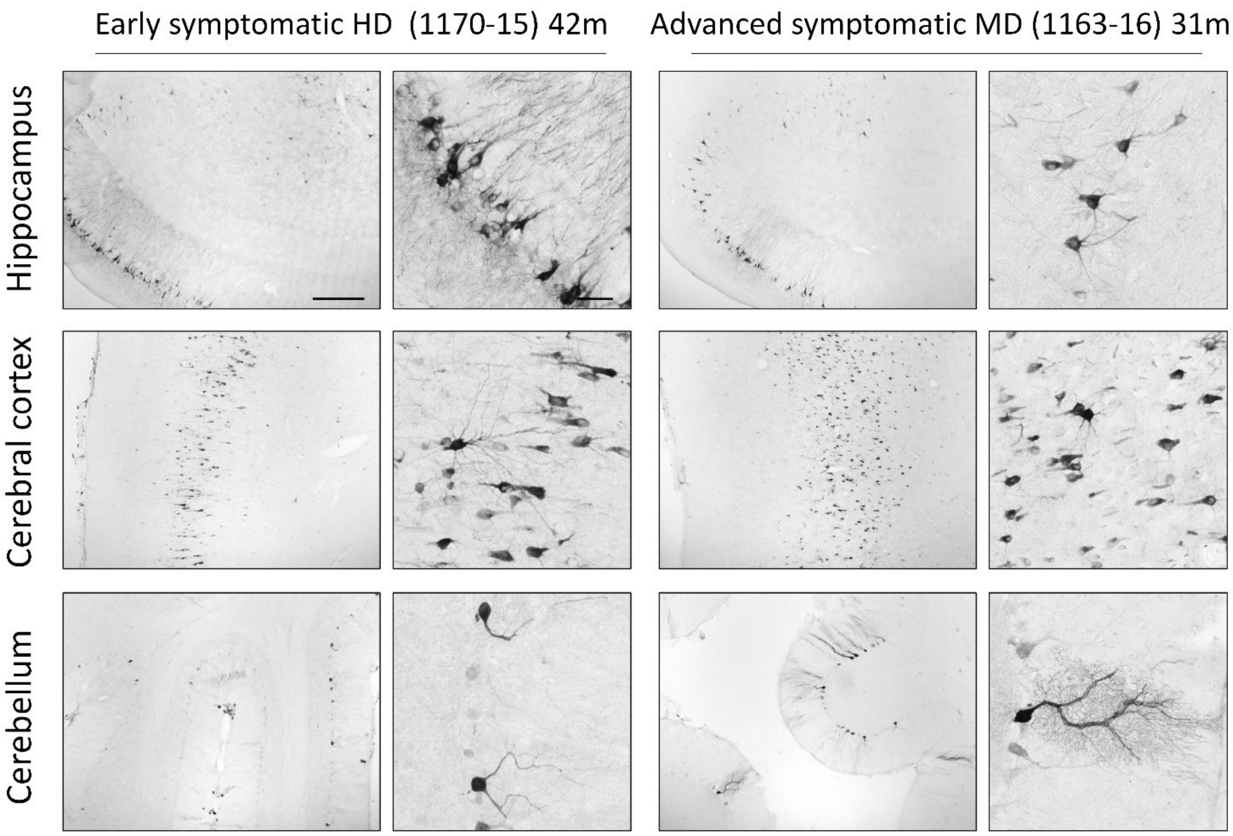

Supplementary Figure 3. Ovine CLN5 protein is expressed in the brains of ICV scAAV9/oCLN5 treated sheep. Representative images of CLN5 immunostaining in brain regions of CLN5$^{-/-}$ sheep treated intracerebroventricularly with scAAV9/oCLN5 at 3 (pre-symptomatic), 6 (early symptomatic), and 9 (advanced symptomatic) months of age were compared with healthy control CLN5$^{+/-}$ and untreated CLN5$^{-/-}$ sheep. Whilst CLN5 expression levels in treated sheep brains did not reach endogenous CLN5$^{+/-}$ levels, scAAV9/oCLN5 treatment generated large numbers of transduced CLN5 positive cells in the hippocampus, cerebral cortex (V1, primary visual cortex shown here) and cerebellum. Higher doses achieved greater transduction efficiency. Scale bar represents 50 µm.

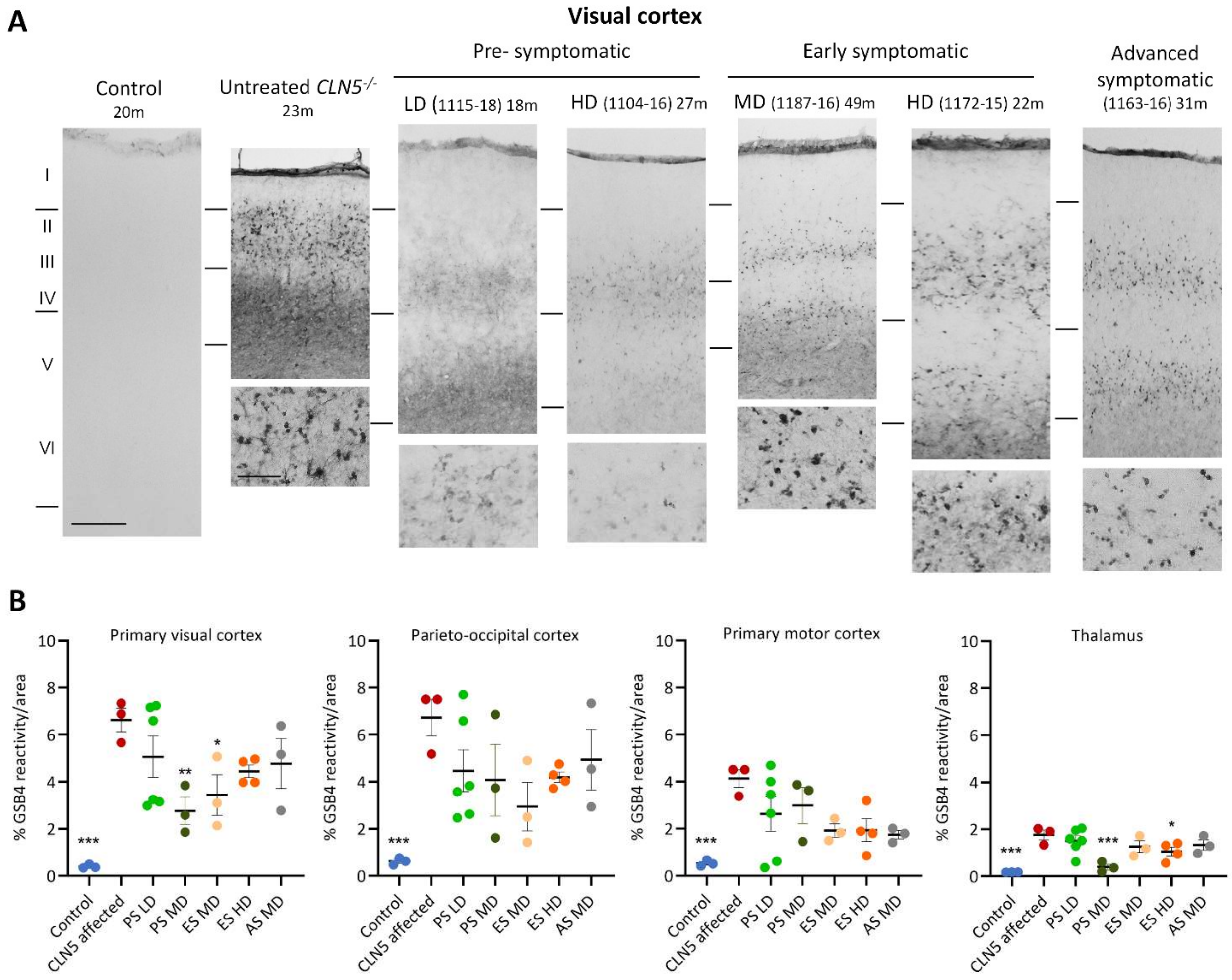

Supplementary Figure 4. ICV scAAV9/oCLN5 attenuates microglial activation. (A) The positive treatment effect on microgliosis can be seen in representative GSB4-stained images of the visual cortex of CLN5$^{-/-}$ sheep treated intracerebroventricularly with scAAV9/oCLN5 at 3 (pre-symptomatic), 6 (early symptomatic), or 9 (advanced symptomatic) months of age when compared with healthy control CLN5$^{+/-}$ and untreated CLN5$^{-/-}$ sheep. The top line marks the layer I/II boundary, middle line indicates the layer IV/V boundary, and lower line denotes the layer VI/white matter boundary. Scale bar represents 200 µm. (B) Quantification of microgliosis in four key brain regions shows the treatment effect for individual animals in each group (PS pre-symptomatic, green; ES early symptomatic, orange; AS advanced symptomatic, grey). Vertical bars indicate +SEM. Significant differences to untreated CLN5$^{-/-}$ are denoted by asterisks (*** $P < 0.001$, * $P < 0.05$). Low dose (LD); moderate (MD); high (HD) dose.

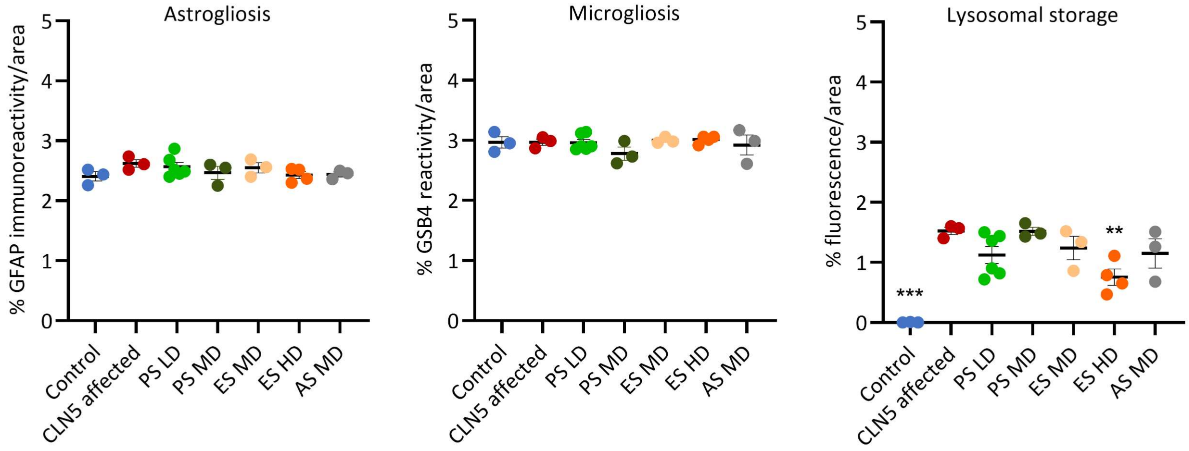

Supplementary Figure 5. ICV scAAV9/oCLN5 attenuates cerebellar lysosomal storage accumulation. Quantification of astrogliosis, microgliosis and lysosomal storage in the cerebellum of healthy control $CLN5^{+/-}$, untreated $CLN5^{-/-}$ sheep and $CLN5^{-/-}$ sheep treated intracerebroventricularly with scAAV9/oCLN5 at 3 (pre-symptomatic), 6 (early symptomatic), or 9 (advanced symptomatic) months of age shows that cerebellar pathology is not a strong disease marker in $CLN5^{-/-}$ sheep. No significant difference was detected for the neuroinflammatory markers, whilst lysosomal storage burden in ICV treated sheep was at similar levels to much younger untreated $CLN5^{-/-}$ sheep and significantly reduced in sheep treated early symptomatically with a high ICV dose (PS pre-symptomatic, green; ES early symptomatic, orange; AS advanced symptomatic, grey). Vertical bars indicate +SEM. Significant differences to untreated $CLN5^{-/-}$ are denoted by asterisks (*** $P < 0.001$, ** $P < 0.01$). Low dose (LD); moderate (MD); high (HD) dose.

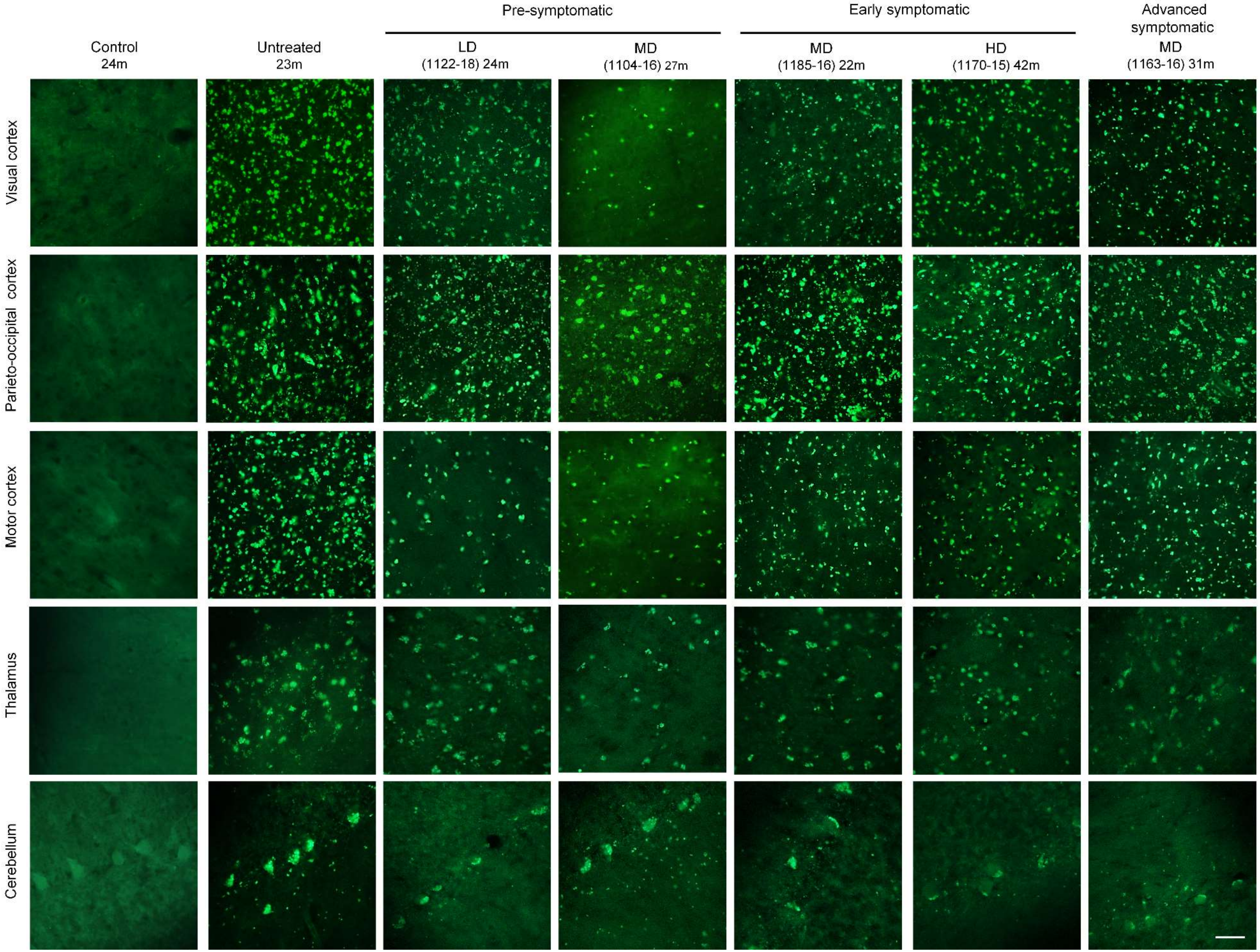

Pre-symptomatic
Early symptomatic
Advanced symptomatic
Control 24m
Untreated 23m
LD (1122-18) 24m
MD (1104-16) 27m
MD (1185-16) 22m
HD (1170-15) 42m
MD (1163-16) 31m
Visual cortex
Parieto-occipital cortex
Motor cortex
Thalamus
Cerebellum

Supplementary Figure 6. ICV scAAV9/oCLN5 slows lysosomal storage accumulation. Representative fluorescent images of storage material in the visual, parieto-occipital and motor cortices, thalamus and cerebellum of $CLN5^{-/-}$ sheep treated intracerebroventricularly with scAAV9/oCLN5 at 3 (pre-symptomatic), 6 (early symptomatic), or 9 (advanced symptomatic) months of age were compared with healthy control $CLN5^{+/-}$ and untreated $CLN5^{-/-}$ sheep. Scale bar represents 50 µm. Low dose (LD); moderate (MD); high (HD) dose.

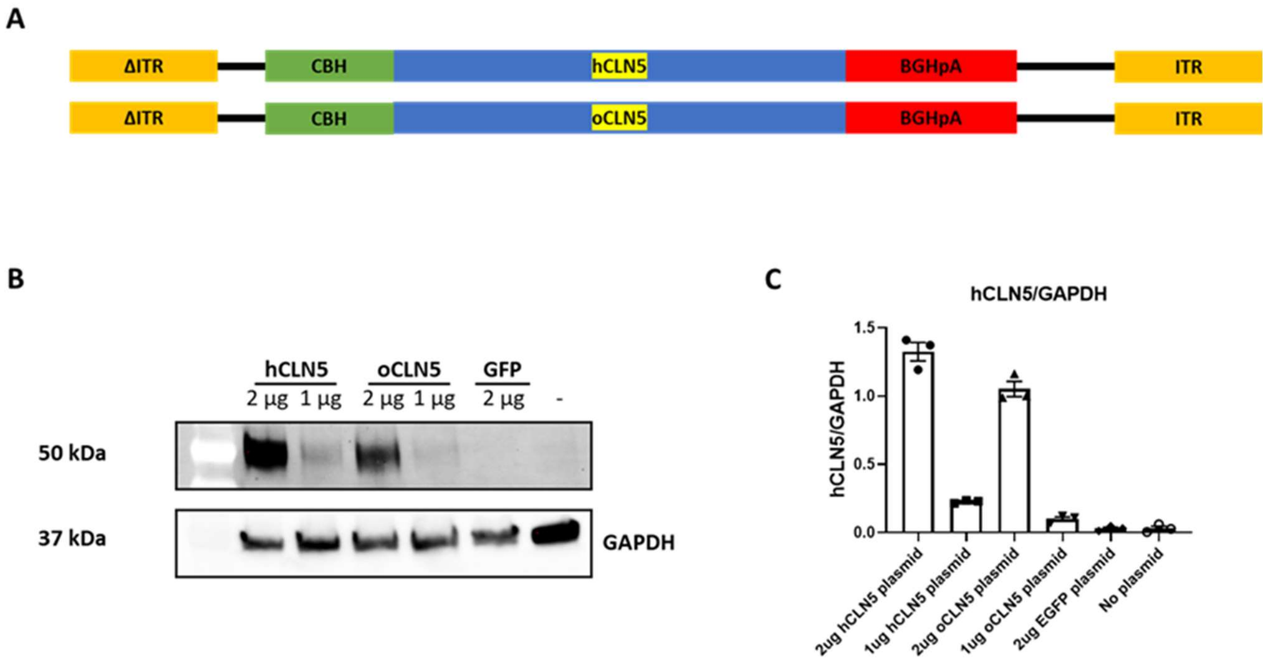

Supplementary Figure 7. Human and ovine CLN5 protein expression is comparable *in vitro*. The protein expression of human (hCLN5) and ovine CLN5 (oCLN5) constructs was assessed by Western blotting. (A) The two main constructs that were used in this study are depicted. (B) A representative Western blot using the anti-CLN5 antibody and the anti-GAPDH antibody (internal control) shows comparable CLN5 protein expression for both constructs. (C) The normalized and aggregated CLN5 expression data from 3 independent experimental replicates indicates no significant difference between the two constructs. Data were compared with an EGFP (enhanced green fluorescent protein) plasmid control and no plasmid. Error bars indicate standard error of the mean.
